# Tunable MEMS-based meta-absorbers for nondispersive infrared gas sensing applications

**DOI:** 10.1038/s41378-024-00851-w

**Published:** 2025-01-08

**Authors:** Kunye Li, Yuhao Liang, Yuxin Liu, Yu-Sheng Lin

**Affiliations:** 1 School of Electronics and Information Technology, Sun Yat-Sen University, 510006 Guangzhou, China; 2 School of Chemistry, Sun Yat-Sen University, 510006 Guangzhou, China; 3 Instrumental Analysis and Research Center, Sun Yat-Sen University, 510275 Guangzhou, China; 4 Sichuan University, 610207 Chengdu, China

**Keywords:** Nanophotonics and plasmonics, Optical sensors, Micro-optics

## Abstract

In conventional nondispersive infrared (NDIR) gas sensors, a wide-spectrum IR source or detector must be combined with a narrowband filter to eliminate the interference of nontarget gases. Therefore, the multiplexed NDIR gas sensor requires multiple pairs of narrowband filters, which is not conducive to miniaturization and integration. Although plasmonic metamaterials or multilayer thin-film structures are widely applied in spectral absorption filters, realizing high-performance, large-area, multiband, and compact filters is rather challenging. In this study, we propose and demonstrate a narrowband meta-absorber based on a planar metal–insulator–metal (MIM) cavity with a metallic ultrathin film atop. Nearly perfect absorption of different wavelengths can be obtained by controlling the thickness of the dielectric spacer. More significantly, the proposed meta-absorber exhibits angle-dependent characteristics. The absorption spectra of different gases can be matched by changing the incident angle of the light source. We also preliminarily investigate the CO_2_ gas sensing capability of the meta-absorber. Afterward, we propose a tunable meta-absorber integrated with a microelectromechanical system (MEMS)-based electrothermal actuator (ETA). By applying a direct current (DC) bias voltage, the inclination angle of the meta-absorber can be controlled, and the relationship between the inclination angle and the applied voltage can be deduced theoretically. The concept of a tunable MEMS-based meta-absorber offers a new way toward highly integrated, miniaturized and energy-efficient NDIR multigas sensing systems.

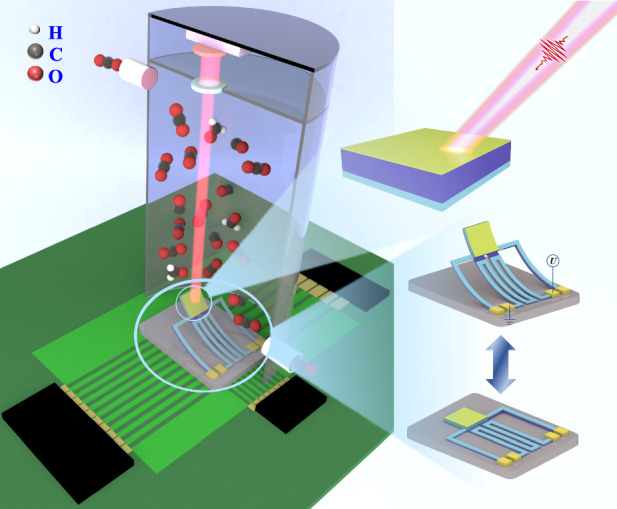

## Introduction

Several molecules have strong absorption characteristics in the mid-infrared (MIR) spectral region from 2 µm to 20 µm, which allows them to be selectively detected^1,2^. On the basis of this molecular “fingerprinting” capacity, the MIR optical gas sensor is a detection technology for tracing multispecies gases. This technology can be utilized to identify and quantify various organic or inorganic substances and offers high sensitivity, high selectivity, and long-term operation stability, which play vital roles in industrial process control, environmental monitoring, and medical diagnosis^3–8^. Optical gas sensors can be categorized according to the presence or absence of light-splitting elements as either dispersive or nondispersive systems. Dispersive gas sensors have excellent resolution and ultrahigh sensitivity for a single absorption line, but they need to be assembled on a desktop and can only be used for special occasions^9,10^. Nondispersive infrared (NDIR) gas sensors analyze the species and concentration of target gases on the basis of the IR absorption characteristics of gas molecules^11^. NDIR gas sensors can achieve parts per million (ppm) sensitivity and reduce the sensor size and cost. Nevertheless, the IR sources of conventional NDIR gas sensors, such as light-emitting diodes (LEDs) and thermal emitters, are broadband, quantum cascade lasers (QCLs) are excessively complicated and expensive, and the IR detector is not selective^12^. To analyze the target gas, a narrowband filter is required to eliminate interference from the nontarget gas. Furthermore, to realize the simultaneous analysis of several target gases in a mixture atmosphere, the multiplexed NDIR gas sensor requires multiple pairs of narrowband filters^13^, which limits the miniaturization and integration of gas sensors. An effective route for the removal of separate filters is to integrate absorption resonance structures with spectral selectivity, such as plasmonic metamaterials or multilayer thin films, into the IR source or detector.

Metamaterials can control and manipulate light‒matter interactions at the subwavelength scale, which can achieve unprecedented optical characteristics, including but not limited to perfect transmission, reflection, and absorption^14–20^. In general, the extraordinary optical characteristics of such artificially designed and manufactured photonic materials are derived from plasmon resonances induced by metallic and dielectric micro/nanostructures with given shapes, sizes, and periodicity^21,22^. It has been shown that perfect absorption can be achieved in a broadband spectrum by designing metamaterials with effective impedance matching with free space^23^. Therefore, a plasmonic metamaterial absorber (PMA) can be regarded as a micro/nanoscale absorption filter. By integrating a PMA into an IR thermal emitter^24–28^ or detector^29–32^ to introduce spectral selectivity, a filter-free NDIR gas sensing system with high sensitivity, high selectivity and compactness can be realized. Consequently, a multiplexed NDIR gas sensing system can be established by designing PMAs of different sizes to match the absorption spectra of different gases^33–35^. Nevertheless, the subwavelength feature of metamaterials requires high-precision micro/nanoscale manufacturing technology, and the multisize PMA arrays also limit the further miniaturization of sensor chips. A multilayer thin-film structure can enhance the absorption resonance by designing an asymmetric metal–insulator–metal (MIM)-based Fabry–Perot (FP) cavity^36–38^, and its application in NDIR gas sensing systems has long been reported, but it can detect only a single gas^39^. Although the resonant wavelength can be matched by designing dielectric layers with different thicknesses or gradient thicknesses^40,41^, this approach either complicates the manufacturing process or requires a special lithography mask. This is not conducive to reducing the cost of the system. On the other hand, the alternative approach is to use the microelectromechanical system (MEMS) technique to enable micro/nanoscale mechanical operation, which has diverse applications in metamaterial-based functional devices. For example, T. Kan et al. presented electromechanically reconfigurable plasmonic devices with a MEMS deformable cantilever and realized the reconfiguration of the spectral response related to the incident angle via an electrostatic driving strategy^42,43^. Advances in MEMS technology enable many actuators to realize in-plane and out-of-plane motions with different electromechanical characteristics according to the requirements of applications. Owing to the diverse electromagnetic properties of PMAs or multilayer thin-film structures, such a hybrid physical sensing platform has advantages in terms of compact feature size and wavelength selective detection^44^.

In this work, we propose and demonstrate an MIR meta-absorber based on a planar MIM cavity. The multilayer thin-film structure does not require a high-precision micro/nanoscale manufacturing process, which results in a lithography-free, large-area, and high-performance absorber. More importantly, the absorption spectra of different gases can be matched by changing the incident angle of the light source, and the sensing capability of the proposed meta-absorber is preliminarily investigated by utilizing CO_2_ gas. Under these conditions, we design a tunable meta-absorber by using a MEMS-based electrothermal actuator (ETA) platform, which possesses a dielectric layer with a single thickness and does not need a special lithography mask. The inclination angle of the meta-absorber can be controlled by applying a direct current (DC) bias voltage on the ETA, and we theoretically deduce the relationship between the inclination angle and the applied voltage. We also design an NDIR multigas sensing system utilizing the proposed tunable MEMS-based meta-absorber to achieve a miniaturized, integrated, and lower-cost gas sensing system.

## Designs and methods

**Fig. 1:**
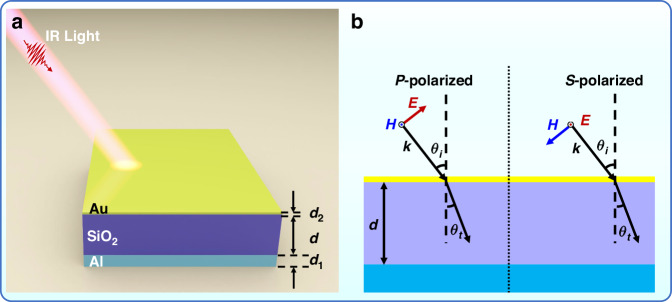
Schematic drawings of 3D and cross-sectional views of the proposed meta-absorber. **a** Meta-absorber is composed of planar Au/SiO_2_/Al cavity. The thicknesses of top and bottom metallic layers are set as *d*_*1*_ = 200 nm and *d*_*2*_ = 10 nm, respectively. **b** The illumination source is incident onto the planar surface with two orthogonal polarization states

Figure 1a shows a schematic of the proposed meta-absorber based on a planar MIM cavity. The top Au ultrathin film is separated from the bottom Al reflective layer with a middle layer of SiO_2_ dielectric thin film. The thicknesses of the three layers are defined as *d*_*1*_, *d*, and *d*_*2*_ from bottom to top. To minimize transmission, the thickness of the Al layer (*d*_1_) is 200 nm so that it is greater than the penetration depth of the IR electromagnetic wave. The thickness of the Au ultrathin film (*d*_2_) is intentionally set to 10 nm to ensure that the IR light can penetrate and form a strong cavity confinement. Arbitrarily polarized light can be expressed as the superposition of two lines of linearly polarized light orthogonal to each other. To facilitate explanation and analysis, we define the polarization state of the electric (*E*) field parallel to the plane of incident light as *P*-polarized and the polarization state of the *E*-field perpendicular to the plane of incident light as *S*-polarized, as shown in Fig. 1b, which are orthogonal linearly polarized light.

**Fig. 2:**
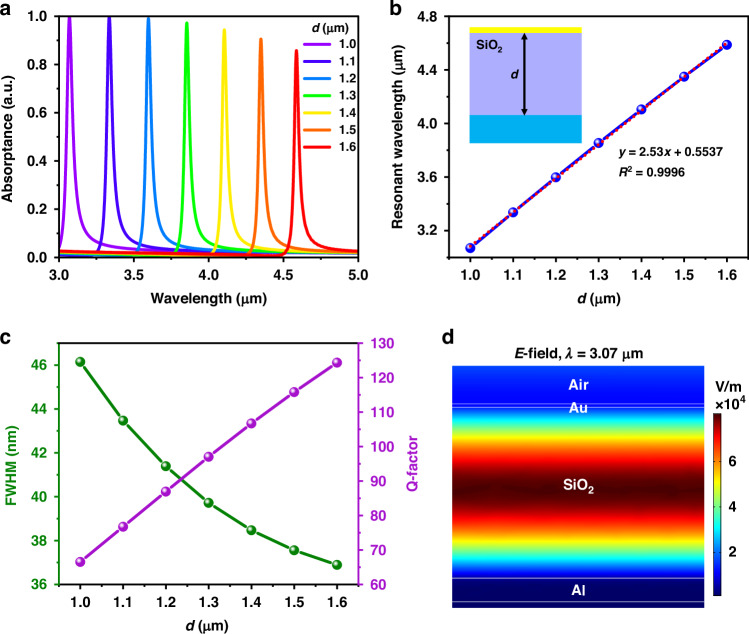
Simulated absorption spectra of the proposed meta-absorber with different d values and the corresponding relationships. **a** Absorption spectra of the meta-absorber when the illumination source is normal incidence onto the planar surface. **b** The corresponding relationship of the resonances and *d* values. **c** The corresponding relationship of FWHM values and Q-factors with *d* values. **d** E-field distribution of the meta-absorber at the resonant wavelength (λ = 3.07 μm) when the *d* value is 1.0 μm

The simulated absorption spectra are obtained by utilizing the full wave finite-difference time-domain (FDTD) method for the meta-absorber based on a planar MIM cavity. The refractive indices of the SiO_2_, Au and Al materials are referred to in the literature^45–48^. Figure 2a shows the simulated absorption spectra of meta-absorbers with different dielectric layer thicknesses (*d*) when the illumination source is normally incident onto a planar surface. For a planar thin-film structure, the meta-absorber is obviously polarization independent when the light is normally incident. As shown in Fig. 2a, when the *d* value increases from 1.0 μm to 1.6 μm, the absorption peak redshifts from 3.07 μm to 4.60 μm. Furthermore, the absorptance reaches 100% when the *d* value increases from 1.0 μm to 1.2 μm and then decreases slightly as the *d* value increases from 1.3 μm to 1.6 μm. Figure 2b shows the relationship between the resonant wavelength and *d* values; there is an excellent positive linear correlation between them, and the linear correction coefficient (*R*^2^) is equal to 0.9996. These results indicate that the desired resonant wavelength can be linearly obtained by simply changing the thickness of the dielectric layer so that the meta-absorber can be applied to a broader frequency band. The outstanding narrowband characteristic is required for NDIR gas sensing applications, which can prevent interference caused by other gases with adjacent absorption bands. The quality (Q) factor is a vital parameter for evaluating the performance of narrowband absorbers and is defined as the resonance divided by the full width at half-maximum (FWHM) bandwidth. Figure 2c shows the corresponding relationships of the FWHM values and the Q-factors with the *d* values. When the *d* value increases evenly from 1.0 μm to 1.6 μm, the FWHM value of the resonance is reduced from 46 nm to 37 nm, and the Q-factor increases from 67 to 124. These data suggest that the performance of the meta-absorber based on a planar MIM cavity is superior to that of other types of narrowband meta-absorbers^24,26,31–33,35,39–41^ in the same wavelength range. To visualize and briefly comprehend the absorption mechanism in the planar MIM resonator, the *E*-field distribution of the meta-absorber at the resonant wavelength is calculated at *d* = 1.0 μm, as shown in Fig. 2d. Since the metallic layer at the bottom is thick enough to be greater than the penetration depth of the electromagnetic wave in the MIR spectrum range, the absorptance (*A*) can be simply calculated as *A* = 1 – *R* – *T* (*T* = 0), where *R* and *T* represent the reflectance and transmittance, respectively. In Fig. 2d, the *E*-field is highly confined to the dielectric layer between two metallic films and then generates a resonance because the incident wave and the reflected wave interfere to form a standing wave. Accordingly, almost no reflection occurs (*R* ≈ 0) at the resonant wavelength, and the absorption reaches a maximum.

**Fig. 3:**
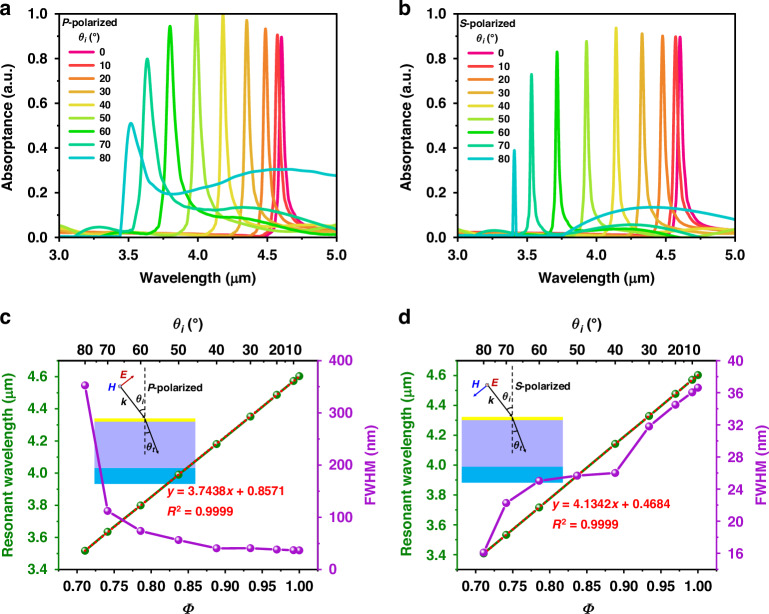
Simulated absorption spectra of the proposed meta-absorber with different incident angles and the corresponding relationships. Meta-absorber with different incident angles at **a** *P*-polarized and **b** *S*-polarized waves. The corresponding relationships of the resonances and FWHM values with *Φ* values at **c** *P*-polarized and **d** *S*-polarized waves

Unlike many other PMAs, the resonance of a meta-absorber based on a planar MIM cavity is usually sensitive to the incident angle of the illumination source, which is a significant inspiration for matching the absorption resonance with different gas absorption spectra. Figure 3a, b shows the simulated absorption spectra of the meta-absorbers with different incident angles (*θ*_*i*_) of *P*-polarized and *S*-polarized waves, respectively, when the dielectric layer thickness is 1.60 μm. The thickness is selected intentionally, which will be discussed later in detail. When the incident angle of the illumination source increases from 0° to 80°, the resonances of the *P*- and *S*-polarized waves are blueshifted from wavelengths of 4.60 μm to 3.52 μm and from wavelengths of 4.60 μm to 3.41 μm, respectively; the tuning ranges are 1.08 μm and 1.19 μm, respectively. Although the absorptance of the meta-absorber decreases at large incident angles, it is still 0.80 and 0.73 for *P*-polarized and *S*-polarized waves, respectively, when the incident angle is 70°. In addition, the high selectivity is directly related to the FWHM value of the absorption resonance. As shown in Fig. 3c, d, for the *P*-polarized wave, the FWHM value increases with increasing incident angle, and the maximum FWHM value is 112 nm, with the exception of at 80° with a large divergence. For the *S*-polarized wave, the FWHM decreases with increasing incident angle, and the maximum FWHM is 37 nm. Therefore, two different gases can be detected and distinguished as long as their resonance difference exceeds 112 nm for *P*-polarized waves and 37 nm for *S*-polarized waves. These results show that the proposed meta-absorber based on a planar MIM cavity has excellent dependence on the incident angle and narrow absorption, which could have potential applications in angle-sensitivity absorbers and directional thermal emitters.

On the other hand, the tuning mechanism of the meta-absorber can be achieved by changing the incident angle, but it is not a linear process. The meta-absorber is essentially an asymmetric FP resonator. Here, “asymmetric” emphasizes that the geometry of the reflective structures is not symmetric and that they are highly reflective metallic layers with different thicknesses. The conventional FP resonator is constructed of two parallel reflectors, and the resonance condition of the meta-absorber is related to the phase shifts caused by multiple round trips of electromagnetic waves in the resonator^49^. The metallic layer is not a perfect electrical conductor, which limits the optical conductivity, and the metallic film atop it is ultrathin, which allows light interaction inside and outside the cavity. Both practical conditions introduce additional optical phase accumulations^50^. Analogous to the FP cavity, the resonance condition of the meta-absorber based on the planar MIM cavity is approximately determined by the following formulas^36^.

1 $$2\beta +{\varphi }_{\text{c}}=2m{{\pi }}$$

2 $$\beta =\frac{2{{\pi }}}{{\lambda }_{{r}}}{{n}}_{{d}}{dcos}\left({{{\theta }}}_{{t}}\right)$$

where *β* is the phase shift of light upon traveling inside the dielectric cavity with the refractive index *n*_*d*_ and the thickness *d*, *φ*_*c*_ is the phase shift due to reflection from the corresponding boundary of the resonance cavity, *m* is a positive integer, *λ*_*r*_ is the resonant wavelength, and *θ*_*t*_ is the included angle between the light propagating in the resonant cavity and the normal, as shown in Fig. 1b. Since the top metallic film is ultrathin and the bottom metallic film is highly reflective, *φ*_*c*_ can be approximately regarded as a constant independent of the fixed angle, whereas its values are generally unequal for *P*-polarized and *S*-polarized waves, which explains their resonance difference^51^ (see Supplementary Note 1). According to Eqs. (1) and (2), we can obtain *λ*_*r*_ ∝ *d* when the illumination source is incident at a certain angle, which is consistent with the results of Fig. 2b. The angular shift caused by the ultrathin metallic film is negligible, so the following relationship can be obtained according to the refraction law.

3 $${n}_{\text{air}}\sin ({\theta }_{i}){=n}_{d}\sin ({\theta }_{t})$$

where *n*_air_ ≈ 1 represents the refractive index of air. Through mathematical relationships, simultaneous Eqs. (1)–(3) can be used to obtain the relationship between the resonance wavelength and incident angle as follows:

4 $${{\rm{\lambda }}}_{\text{r}}\propto \sqrt{1{-}{\left(\frac{\sin ({{{\theta }}}_{{i}})}{{{n}}_{{d}}}\right)}^{2}.}$$

To verify the theoretical relationship, we define the parameter $\varPhi =\sqrt{1{-}{(\sin ({\theta }_{i})/{\text{n}}_{d})}^{2}}$ and obtain the refractive index of SiO_2_ as 1.39 from the literature^45^ (see Supplementary Note 2). Figure 3c, d shows the corresponding relationships of the resonances and *Φ* values under *P*-polarized waves and *S*-polarized waves, respectively. Although the variation rate of the *P*-polarized wave is less than that of the *S*-polarized wave, the resonances and *Φ* values of both have high linear correlations, with *R*^2^ = 0.9999. Therefore, the above theoretical relation is perfectly demonstrated.

**Fig. 4:**
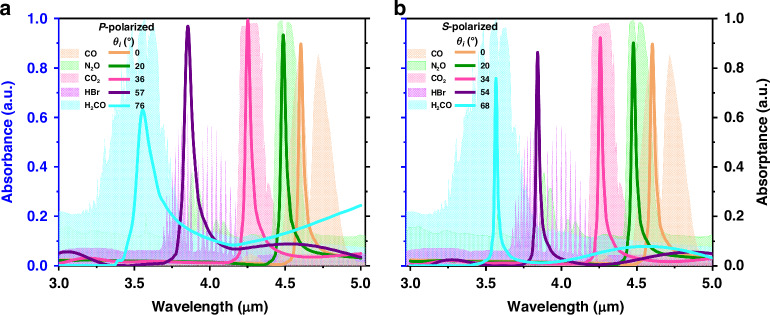
Absorption spectra of different gases correspond to the specific spectra of the proposed meta-absorber. Meta-absorber with different incident angles at **a** *P*-polarized and **b** *S*-polarized waves (The left axis represents the normalized absorbance of gases, and the right axis represents the absorptance of meta-absorber.)

To elucidate the development potential of the angular tuning capability of the meta-absorber based on the planar MIM cavity in the NDIR gas sensing application, the incident obliquity of *P*-polarized and *S*-polarized waves corresponding to the absorption spectra for five gases, i.e., CO, N_2_O, CO_2_, HBr, and H_2_CO obtained from the NIST Chemistry WebBook^52^, are calculated, as shown in Fig. 4a, b, respectively. These results are in accordance with the theoretical relation obtained above and the linear expressions in Fig. 3c, d. The thickness of the dielectric layer is 1.60 μm since the resonant wavelength of the meta-absorber matches the absorption wavelength of CO gas at 4.60 μm when the illumination source has a normal incidence, according to the results in Fig. 2b. A comparison of the results in Fig. 4a with those in Fig. 4b reveals that the absorption spectra of the meta-absorber for the *S*-polarized wave have a narrower FWHM value and better absorption characteristics at a larger incident angle than those for the *P*-polarized wave. The demonstrated absorption spectra of *P*-polarized and *S*-polarized samples at different incident angles allow the capture of the IR signature of the target gas and the rejection of other dense IR absorption spectra, which benefits from the outstanding narrowband absorption and angular tuning of the proposed meta-absorber.

**Fig. 5:**
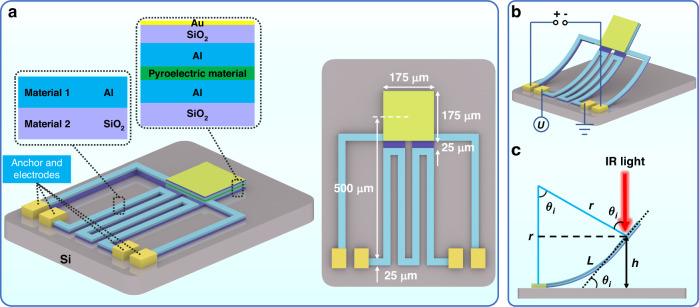
MEMS-based meta-absorber and the calculation model. **a** Schematic drawings of MEMS-based meta-absorber and the geometrical denotations, **b** MEMS-based meta-absorber after releasing the cantilevers, and **c** calculation model of cross-sectional cantilever

However, changing the incident angle of the light source is a difficult problem in integrated and miniaturized gas sensor chips. The same effect can be satisfied by using MEMS technology to realize the out-of-plane movement of the meta-absorber to change the inclination angle. As shown in Fig. 5a, we propose a MEMS-based meta-absorber design, which is composed of a meta-absorber based on a planar MIM cavity integrated with an ETA platform. The role of the pyroelectric layer is to convert the light energy absorbed by the meta-absorber to output electrical signals for detection. The Al layers on both sides of the pyroelectric material are used as thermoelectric voltage sensing layers, which are connected with two electrodes on the silicon (Si) substrate through Al wires. Simultaneously, the upper Al layer serves as the reflective layer of the meta-absorber, and the lower Al layer serves as the metallic layer of the ETA. The ETA is a bilayer composed of SiO_2_ and Al materials on a Si substrate, and the area of the meta-absorber is 175 μm × 175 μm, which is connected and insulated with an ETA platform through a SiO_2_ dielectric layer. The line width of the ETA is kept constant at 25 μm, and the distance from the top of the anchor to the center of the meta-absorber is 500 μm. The comprehensive fabrication process flow of the MEMS-based meta-absorber is described in Supplementary Note 3. Figure 5b shows a schematic of the MEMS-based meta-absorber after the bilayer cantilever is released; such out-of-plane deformation is achieved by the residual stress in the bilayer cantilever. Owing to the temperature variation in the fabrication process, the strong mismatch between the thermal expansion coefficients (TECs) of SiO_2_ and Al materials results in the accumulation of residual stress in the structure. When the cantilever is wholly released, this stress manifests as out-of-plane deformation of the ETA, which drives the meta-absorber at the front end to tilt. This prestressed bilayer is essentially a bimorph thermal actuator, and the out-of-plane motion can be tuned by applying a DC bias voltage to the electrodes of the ETA. The surface electric current flow induces resistance heat and then increases the temperature above room temperature. Afterward, the cantilevers could be bent toward the substrate. When the applied voltage is turned off, the cantilever returns to the initial state. Therefore, the radius of curvature of the ETA is determined by both the initial curling and the temperature change through the electric current flow, which can be expressed as

5 $$\frac{1}{r}=\frac{1}{{r}_{0}}-\frac{1}{{r}_{T}}$$

where *r* is the actual radius of curvature, *r*_0_ is the initial radius of curvature, and *r*_*T*_ is the radius of curvature due to the temperature change. By simplifying the calculation model of the cantilever shown in Fig. 5c, *r*_*T*_ can be readily derived as^53^

6 $$\frac{1}{{r}_{T}}=\frac{3}{2}\cdot\frac{\triangle T({\alpha }_{1}-{\alpha }_{2})({t}_{1}+{t}_{2})}{{t}_{1}^{2}+{t}_{2}^{2}+\frac{3}{2}{t}_{1}{t}_{2}+\frac{1}{4}\left(\frac{{E}_{1}{t}_{1}^{3}}{{E}_{2}{t}_{2}}+\frac{{E}_{2}{t}_{2}^{3}}{{E}_{1}{t}_{1}}\right)}$$

where subscripts 1 and 2 represent different materials of the bilayer thin film shown in Fig. 5a, t is the thickness of the deposited layer, *α* is the TEC, ∆*T* is the temperature change on the cantilever, and *E* is Young’s modulus. The *E* values of both the Al and SiO_2_ materials are 70 GPa, and the TEC values are 23.1 × 10^-6^ K^−1^ and 0.5 × 10^−6^ K^−1^ ^54^, respectively.

The relationship between the bending angle of curvature or incident angle (*θ*_*i*_ in Fig. 5c) and the applied voltage can be deduced theoretically. The Joule heat (*q*) induced by the electric current is expressed as Eq. (7) according to Joule’s law.

7 $$q=\frac{{U}^{2}}{{R}_{0}}$$

where *U* is the applied DC bias and *R*_0_ is the resistance of the Al thin film in the ETA. It can be approximately considered that the generated resistance heat is preferentially absorbed by the ETA, and the endothermic heating formula is expressed as

8 $${q}^{{\prime} }={k}_{0}q={cm}\triangle T$$

where $q^{\prime}$ denotes the part of the generated heat absorbed by the ETA, *k*_0_ is a proportionality constant (0 < *k*_0_ < 1), *c* is the specific heat capacity, and *m* is the mass of the ETA. In addition, according to the geometric relation in Fig. 5c, the radius of curvature can be obtained as

9 $$r=\frac{L}{{{\theta }}_{i}}$$

where *L* is the distance from the top of the anchor to the center of the meta-absorber. According to Eqs. (5)–(9), the relationship between *θ*_*i*_ and *U* can be derived as *θ*_*i*_ ∝ – *U* ^2^. To verify and quantify the theoretical relationship, we define *θ*_*i*_ = *π*/2 for the maximum incident angle when *U* is equal to 0 volts and define *θ*_*i*_ = 0 for the minimum incident angle when *U* is equal to *U*_*m*_; *θ*_*i*_ is expressed in radians here. The actual *U*_*m*_ is a measured value that needs to be determined by experiments, but it can be approximated from the simulation only to verify the theoretical relation. Thus, the ultimate relationship is obtained as

10 $${\theta }_{i}=\frac{{{\pi }}}{2}\left(1-\frac{{U}^{2}}{{U}_{m}^{2}}\right)$$

On the other hand, the vertical displacement (*h* in Fig. 5c) of the ETA is often obtained more directly than the curvature angle according to the geometric relation in Fig. 5c. This can be expressed as

11 $$h=r\left(1-\cos \left({\theta }_{i}\right)\right)$$

To verify Eq. (10), the whole MEMS-based meta-absorber is modeled and simulated via the finite element simulation software COMSOL Multiphysics. The thicknesses of both the Al and SiO_2_ layers in the ETA are 3 μm. To simulate the process of releasing stress, a positive prestress value is set for the Al layer with a large TEC in the state of tensile residual stress, and a negative prestress value is set for the SiO_2_ layer with a small TEC in the state of compressive residual stress.

## Results and discussion

**Fig. 6:**
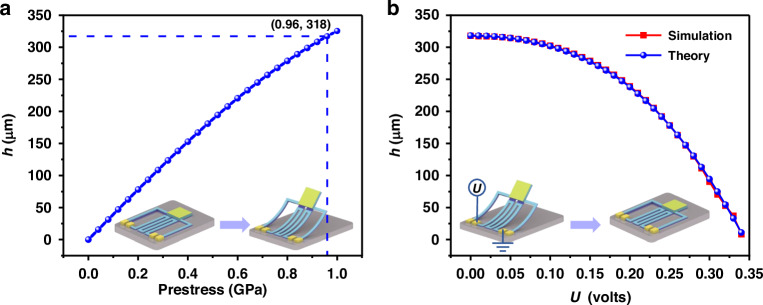
Relationships of the prestress and *U* values to *h* values. **a** Prestress to *h* curve. **b** *U* to *h* curve

Figure 6a shows the corresponding relationship between the *h* values and the prestress values of the ETA, which is the process of releasing the cantilever. More residual stresses accumulate in the fabrication process, resulting in a greater displacement height after the structure is released. The *L* value is 500 μm, and the elevation height of the meta-absorber center is approximately 318 μm when the angle of curvature is *π*/2 according to Eqs. (9) and (11). The cumulative residual stress is approximately 0.96 GPa. By applying a DC bias to the electrodes of the ETA, Joule heat is generated, and the temperature of the cantilever increases to make the ETA move downward. Figure 6b shows the corresponding relationship between the *h* values and the magnitude of the bias voltage (*U*). The red dotted line represents the data obtained via simulation, and accordingly, the value of *U*_*m*_ is approximately 0.345 volts. The relationships of current flow and power with the applied DC bias voltage are plotted in Fig. S5 in Supplementary Note 4. These results show that the electrothermal drive strategy has excellent mechanical deformation performance and an ultralow power consumption configuration. The blue dotted line represents the data calculated via Eqs. (10) and (11), which agree well with the simulation data. On the basis of these results, the theoretical relation *θ*_*i*_ ∝ – *U*^2^ is sufficiently verified. Moreover, the designed ETA structure can completely actuate the front-end multilayer structure but hardly degrades its theoretical mechanical performance. Consequently, the inclination angle of the MEMS-based meta-absorber can be changed by driving different input DC bias voltages, and the incident angle of the illumination source can be actively controlled. With the combination of Eq. (4) and the results in Fig. 3c, d, the homologous incident angle can be calculated according to the absorption wavelength of the target gas, and the corresponding driving voltage can be obtained via an inverse solution. Therefore, an actively tunable meta-absorber for gas sensing applications can be realized.

**Fig. 7:**
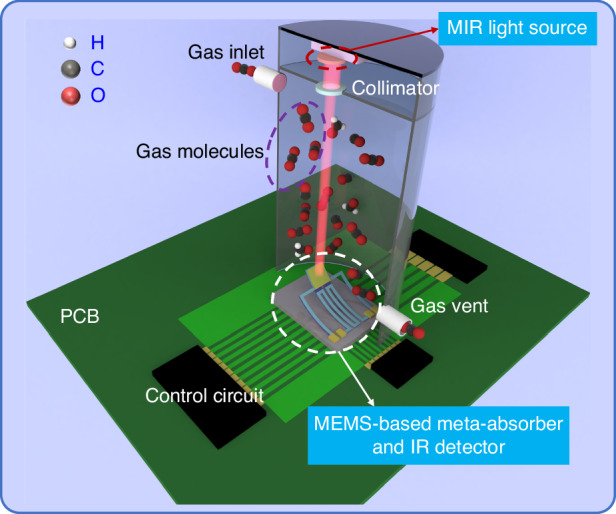
Schematic of the multigas sensing system based on the NDIR architecture with MEMS-based meta-absorbers enabling narrowband IR absorption. The system is composed of three parts: broadband MIR light sources with necessary collimating and focusing optics, gas cells, and MEMS-based meta-absorbers integrated with an IR detector bonded on a PCB

On the basis of the above results, an NDIR multigas sensing system is proposed, as shown in Fig. 7. The proposed multigas sensing system based on the NDIR architecture with a MEMS-based meta-absorber enables tunable narrowband IR absorption, which is composed of three parts: broadband MIR light sources with necessary collimating and focusing optics, gas cells, and MEMS-based meta-absorbers integrated with an IR detector. The integrated meta-absorber and IR detector chip is bonded on a carrier printed circuit board (PCB) in a chip-on-board configuration. The collimated MIR light is directly incident on the surface of the MEMS-based meta-absorber. The necessary collimation accuracy for NDIR gas sensing is interpreted in Supplementary Note 5. The role of the meta-absorber is to absorb the light at the resonant wavelength and convert the radiant energy to thermal energy. The pyroelectric material and the Al layers on both sides form an IR detector, which converts thermal energy to electrical energy in the form of an electromotive force. This voltage signal can then be detected by an external integrated circuit, and the voltage amplitude correlates with the gas concentration. The meta-absorber absorbs the most radiant energy at the resonant wavelength when there is no target gas to be detected. Thus, the voltage signal amplitude obtained by detection at this time is the largest. When the target gas has a characteristic absorption peak matched with the resonance wavelength, the radiation energy reaching the meta-absorber is reduced since the target gas absorbs a part of the light energy, which causes weakening of the output voltage signal. According to the Beer‒Lambert law, the higher the concentration of the target gas is, the greater the change in voltage amplitude (see Supplementary Note 10 for details). Since the resonant wavelength of the meta-absorber based on the planar MIM cavity is angle dependent, the incident angle can be indirectly controlled by applying a specific driving voltage to the ETA, which filters absorbed radiation to the spectral absorption band of the various target gases, as shown in Fig. 4. This renders additional pairs of narrowband filter elements in conventional NDIR gas sensors obsolete, and a filter-free multiplexed NDIR gas sensing architecture can be achieved.

To preliminarily verify the angle-dependent characteristic and validity of gas sensing of the meta-absorber based on a planar MIM cavity, we fabricated a meta-absorber with a dielectric layer thickness of 1.46 μm to measure its angle-dependent characteristic. The fabrication of the narrowband meta-absorber starts from the deposition of 10-nm-thick Cr (adhesive layer) and 200-nm-thick Al on a polished Si wafer sequentially via an electron-beam (E-beam) evaporation process (model: DE400). SiO_2_ with a thickness of 1.46 μm was then deposited via an inductively coupled plasma chemical vapor deposition (ICP-CVD) process (model: PlasmaPro System100 ICP180-CVD). Finally, 5 nm Cr and 10 nm Au were deposited via the E-beam evaporation process. This meta-absorber fabrication process is a lithography-free process that is easy to manufacture and cost-effective.

**Fig. 8:**
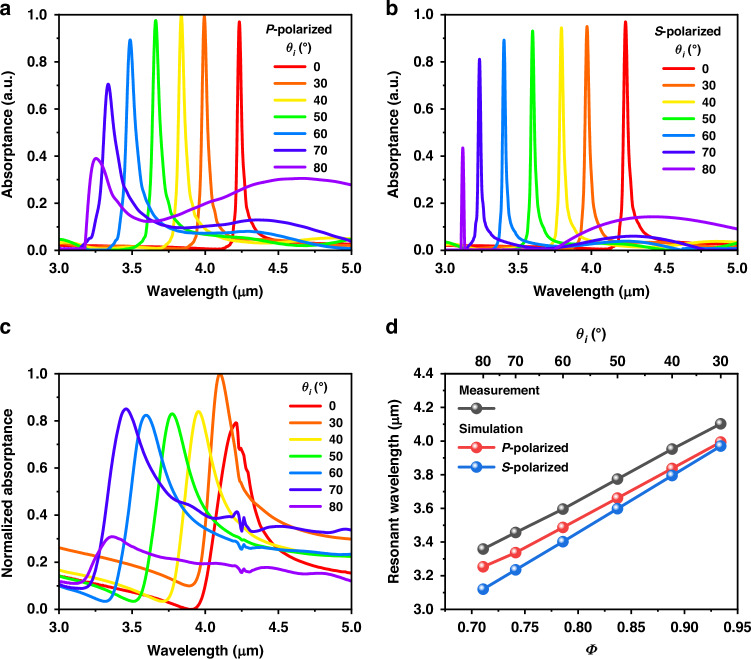
Simulated and measured absorption spectra of the meta-absorber under the condition of *d* = 1.46 μm. Simulated spectra of the meta-absorber operated at **a** *P*-polarized and **b** *S*-polarized waves with different incident angles, respectively. **c** The measured normalized absorption spectra of the meta-absorber at different incident angles. **d** The corresponding relationships of the resonances and *Φ* values obtained by the simulated and measured results

Figure 8a, b shows the simulated absorption spectra of the meta-absorber (*d* = 1.46 μm) for *P*-polarized waves and *S*-polarized waves, respectively, with different incident angles. The resonance is at a wavelength of 4.24 μm when the incoming light has a normal incidence (*θ*_*i*_ = 0°), which is consistent with the trend shown in Fig. 2b. The variation tendency of the absorption spectra is analogous to that in Fig. 3a, b when the incident angle increases from 0° to 80°. For *P*-polarized waves, the resonances are blueshifted from wavelengths of 4.24 μm to 3.25 μm. For the *S*-polarized wave, the resonances are blueshifted from wavelengths of 4.24 μm to 3.12 μm. The optical absorptance of the fabricated meta-absorber was characterized via reflectance measurements via a combined Fourier transform infrared (FTIR) spectrometer-IR microscope (model: Nicolet 6700-Continuμm). The measured spectra are shown in Fig. 8c. The light source used in the FTIR spectrometer is unpolarized, and the measurement data of meta-absorbers with incident angles of 10° and 20° cannot be measured due to the limitation of the incident angle set by the objective lens of the FTIR microscope. For the measured absorption spectra, the resonances are blueshifted from wavelengths of 4.21 μm to 3.35 μm by changing the incident angle from 0° to 80°. These results almost match the simulation results. Notably, the spectral fluctuation around the wavelength of 4.25 μm is derived from the interference of CO_2_ gas in the atmosphere. Figure 8d shows the corresponding relationships of the resonances and *Φ* values obtained from the simulated and measured results. The measurement results also show angular dependence. The measured resonant wavelengths and absorption bandwidths slightly deviate from those of the simulation when the incident angle increases due to the unpolarized light source (see Supplementary Note 6), the limited resolution of the FTIR spectrometer, and the artificial error in the actual incident angle. However, the more substantial and essential reason is that the actual refractive index deviates from the simulation value because the manufacturing process deviates in terms of the thickness and quality of the SiO_2_ and Au thin films. The influence of the thickness of the Au thin film on the absorption spectra is explained in Supplementary Note 7. These deviations can be alleviated by using an FTIR spectrometer with a higher resolution polarized light source, controlling the process conditions, and making the incident angle strictly and accurately. Nevertheless, the relationships of the resonances and *Φ* values are quite linear. These results suggest the angle-dependent characteristic of the meta-absorber based on the planar MIM cavity, which also guarantees the feasibility of a tunable MEMS-based meta-absorber and multiplexed NDIR gas sensing system.

**Fig. 9:**
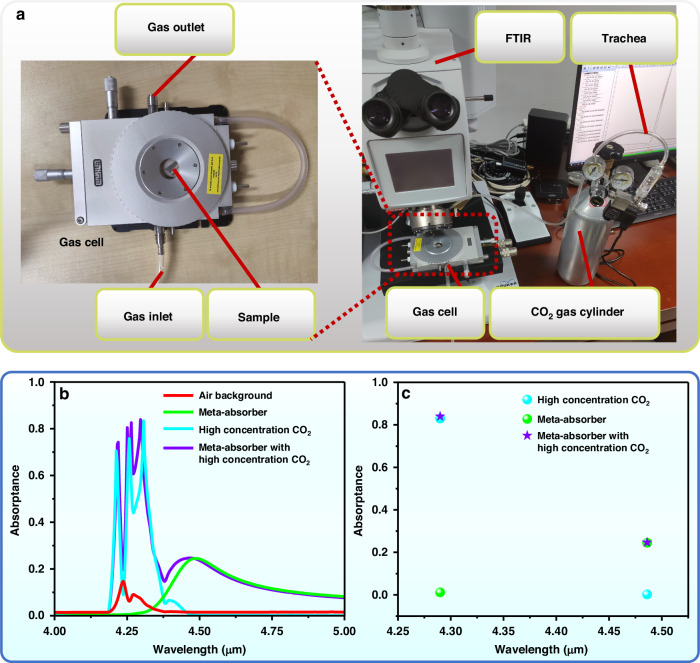
Testing setup and measurement results of CO_2_ gas. **a** Photographs of the testing setup. **b** The measured absorption spectra of the proposed meta-absorber for detecting CO_2_ gas. **c** The distributions of absorption at the wavelengths of 4.29 and 4.48 μm

The gas sensing capability of the meta-absorber based on a planar MIM cavity is preliminarily tested. To clearly observe the influence of high-concentration CO_2_ gas on the absorption spectrum of the meta-absorber, a meta-absorber with a resonance absorption wavelength slightly longer than the CO_2_ gas absorption peak is fabricated according to the relation in Fig. 2b, which has a dielectric layer thickness of 1.55 μm. Figure 9a shows photographs of the test setup. The sample cavity is encapsulated in a gas cell with a gas inlet and outlet, and the gas inlet is connected to a CO_2_ gas cylinder through a tube. The optical absorptance is characterized by the reflectance measurements of the FTIR spectrometer-IR microscopic imaging combined instrument (model: Vertex70-Hyperion3000), and all measurements are launched under the condition of normal incidence of the light source. The reflection spectrum of the air background is calibrated as the reference spectrum by measuring an Al reflective mirror. The detailed testing process is presented in Supplementary Note 8. Figure 9b shows the measured absorption spectra of the meta-absorber for the CO_2_ gas absorption test. The red curve represents the absorption of the air background, and the CO_2_ gas inherent in the air causes the absorption shape around the wavelength of 4.25 μm. Two obvious absorption peaks are observed at wavelengths of 4.24 μm and 4.27 μm. The green curve represents the absorption of the manufactured meta-absorber, and its absorption peak is located at a wavelength of 4.48 μm far away from CO_2_ gas absorption, which is consistent with the prediction. Then, the Al reflective mirror is placed in the sample cavity, and the gas valve of the gas cylinder is opened to measure the absorption of the introduced high-concentration CO_2_ gas, as shown by the cyan curve in Fig. 9b. The introduced high-concentration CO_2_ gas causes strong absorption around the wavelength of 4.25 μm, which is much greater than the absorption of CO_2_ gas inherent in the air. Three obvious absorption peaks are detected at wavelengths of 4.21 μm, 4.26 μm, and 4.29 μm. The reason for the multiple peaks is that the absorption spectrum is supersaturated when CO_2_ gas at high concentrations is introduced, which results in the splitting of absorption peaks at wavelengths of 4.24 μm and 4.27 μm (see Supplementary Note 9 for details). The Al reflective mirror is replaced by a meta-absorber, and the change in the absorption spectrum of the meta-absorber under high-concentration CO_2_ gas is measured, as shown by the purple curve in Fig. 9b. The absorption peak at a wavelength of 4.48 μm is the natural peak of the meta-absorber. In addition, several strong absorption peaks are introduced at wavelengths of 4.21 μm, 4.25 μm, 4.26 μm, and 4.29 μm because of the presence of high concentrations of CO_2_ gas. The absorption spectrum of the meta-absorber (purple curve) measured under high-concentration CO_2_ gas can be regarded as the “combination” of the high-concentration CO_2_ gas absorption spectrum (cyan curve) and the meta-absorber absorption spectrum (green curve). The shape difference around the wavelength of 4.25 μm between the purple curve and the cyan curve is due to the difficulty in ensuring the consistency of the introduced CO_2_ gas concentration over two cycles and the influence of the spectrometer state. Figure 9c shows the absorptance at wavelengths of 4.29 μm and 4.48 μm. At a wavelength of 4.29 μm, where CO_2_ gas has strong absorption, the absorptance of the meta-absorber is very different from that of the meta-absorber with high-concentration CO_2_ gas because of the introduction of high-concentration CO_2_ gas, which increases from 0.012 to 0.840. In contrast, at a wavelength of 4.48 μm from CO_2_ gas absorption, the absorptance of the meta-absorber is 0.25 and changes little even with a high concentration of CO_2_ gas. These experimental results preliminarily verify the influence of CO_2_ gas on the absorption spectrum of the meta-absorber. In addition, the theoretical feasibility of NDIR gas sensing has also been fully explained to support the idea in Fig. 7 after spectral verification (see Supplementary Note 10). It is reasonable to believe that the proposed meta-absorber is effective in NDIR gas sensing applications after the IR detector is integrated.

## Conclusion

In conclusion, we propose and demonstrate a meta-absorber based on a planar MIM cavity, which combines the merits of being lithography-free and having a large area and high performance. The proposed device shows optical angle-dependent characteristics and feasibility for NDIR gas sensing applications when CO_2_ gas is used as the testing target. By integrating MEMS-based ETA, we also propose an actively tunable meta-absorber to eliminate multiple pairs of narrowband filters and optimize the existing multiplexed NDIR gas sensor chip. Owing to the limitations of experimental conditions, the work of combining MEMS-based, tunable meta-absorbers and integrating IR detectors to measure different varieties and concentrations of gases will proceed in the future. Our work potentially opens a novel avenue for a new generation of highly integrated, miniaturized, and low-cost NDIR gas sensors. Moreover, owing to the universality of the basic principle, a MEMS-based, tunable meta-absorber with great flexibility and high-throughput manufacturing convenience is suitable for a broad spectrum and can be further applied in angle sensing, thermal emission engineering, and imaging system applications.

## Supplementary information

Tunable MEMS-based meta-absorber for non-dispersive infrared gas sensing applications

## Data Availability

Data will be made available upon request.
