# Supplementary material for: Tunable MEMS-based meta-absorbers for nondispersive infrared gas sensing applications
Source: Microsyst Nanoeng. 2025 Jan 8;11:2. doi: 10.1038/s41378-024-00851-w (PMC11707267; doi:10.1038/s41378-024-00851-w)
Supplement: Supplementary file 1 — Tunable MEMS-based meta-absorber for non-dispersive infrared gas sensing applications [file 41378_2024_851_MOESM1_ESM.docx]

**Supplementary Information**

Tunable MEMS-based meta-absorber for non-dispersive infrared gas sensing applications

Kunye Li^1†^, Yuhao Liang^2,3†^, Yuxin Liu^1^, and Yu-Sheng Lin^4*^

^1^School of Electronics and Information Technology, Sun Yat-Sen University, Guangzhou, 510006, China.

^2^School of Chemistry, Sun Yat-Sen University, Guangzhou, 510006, China.

^3^Instrumental Analysis and Research Center, Sun Yat-Sen University, Guangzhou, 510275, China.

^4^Sichuan University - Pittsburgh Institute, Chengdu, 610207, China.
^†^These authors contributed equally; *E-mail: yusheng.lin@scupi.cn

Supplementary Note 1

**The explanation of resonance difference between *P*- and *S*-polarized resonant wavelengths in Fig. 3.**

To explain the difference between *P*- and *S*-polarized resonant wavelengths, the resonance condition of the meta-absorber based on planar MIM cavity is approximately determined by the following formulas [1].

|  | $\text{2}\text{β}\text{ +}\text{ φ}_{\text{c}}\text{ = 2}\text{mπ}$ | (S1) |
| --- | --- | --- |
|  | $\text{β }\text{= }\frac{\text{2}\text{π}}{\text{λ}_{\text{r}}}\text{n}_{\text{d}}\text{d}\text{cos(}\text{θ}_{\text{t}}\text{)}$ | (S2) |

where *φ_c_* is the phase shift due to the reflection from the corresponding boundary of the resonant cavity. Considering that light is incident on the bottom metal surface at *θ_t_*, the reflection coefficients of *P*-polarized and *S*-polarized waves are as follows according to Fresnel formulas [2].

|  | $\text{r}_{\text{p}}\text{ =}\frac{\text{tan(}\text{θ}_{\text{t}}-\text{θ}_{\text{t}\text{2}}\text{)}}{\text{tan(}\text{θ}_{\text{t}}+\text{θ}_{\text{t}\text{2}}\text{)}}$ | (S3) |
| --- | --- | --- |
|  | $\text{r}_{\text{s}}\text{ =}-\frac{\text{sin(}\text{θ}_{\text{t}}-\text{θ}_{\text{t}\text{2}}\text{)}}{\text{sin(}\text{θ}_{\text{t}}+\text{θ}_{\text{t}\text{2}}\text{)}}$ | (S4) |
|  | $\text{sin(}\text{θ}_{\text{t}\text{2}}\text{) = }\frac{\text{n}_{\text{d}}}{\tilde{\text{n}_{\text{m}}}}\text{sin(}\text{θ}_{\text{t}}\text{)}$ | (S5) |

where *θ_t_*_2_ is refraction angle, and $\tilde{\text{n}_{\text{m}}}$ is complex refractive index of metal. Since $\tilde{\text{n}_{\text{m}}}$ is a complex number, *θ_t_*_2_ , *r_p_* and *r_s_* are also complex numbers, and *θ_t_*_2_ no longer has the meaning of the conventional refraction angle. The complex reflection coefficients indicate that the reflected *P*-polarized and *S*-polarized waves have phase shifts relative to the incident waves, and the phase shifts of *P*-polarized and *S*-polarized waves are generally different, and their values are determined by the following formulas.

|  | $\text{φ}_{\text{cp}}=\text{arg(}\text{r}_{\text{p}}\text{)}$ | (S6) |
| --- | --- | --- |
|  | $\text{φ}_{\text{cs}}=\text{arg(}\text{r}_{\text{s}}\text{)}$ | (S7) |

where arg(*r*) indicates the argument of a complex number *r*. The subscript labels *p* and *s* are *P*- and *S*-polarized waves, respectively. As shown in Figs. S1(a) and (b), we calculate the *φ_cp_*_1_ and *φ_cs_*_1_ values when the light is reflected at the interface between SiO_2_ and Al layers by using MATLAB software according to Eqs. (S3-S7), where the refractive index *n_d_* of SiO_2_ as 1.39 according to Supplementary Note 2 and the complex refractive index $\tilde{\text{n}_{\text{m}}}$ of Al is obtained from the simulation software Ansys Lumerical FDTD and literature [3].

As can be seen from Fig. S1, *φ_cp_*_1_ and *φ_cs_*_1_ are unequal, *φ_cp_*_1_ are positive numbers while *φ_cs_*_1_ are negative numbers. The reflection at the interface between SiO_2_ and Au layers also has similar difference in phase shifts of *P*-polarized and *S*-polarized waves, which results in the *S*-polarized resonances are different (slightly smaller) than *P*-polarized resonances even at the same incident angle.


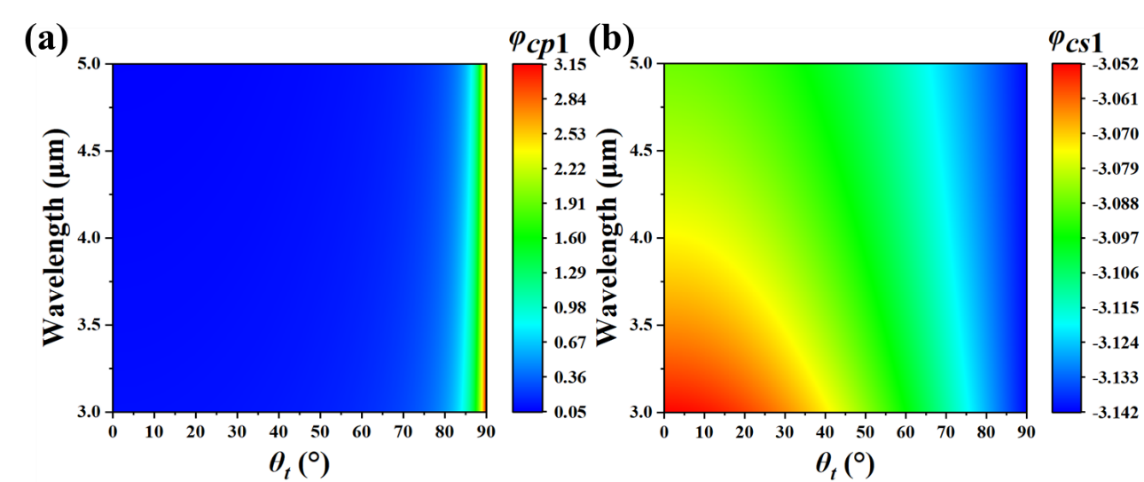


Fig. S1. The values of (a) *φ_cp_*_1_ and (b) *φ_cs_*_1_ when incident light is reflected at the interface between SiO_2_ and Al layers.

Supplementary Note 2

**The complex refractive index data of SiO_2_ material.**

For the wavelength-dependence of the SiO_2_ refractive index, the material database is used for simulation fitting, as shown in Figs. S2(a) and (b). The material data comes from Palik's handbook of optical constants of solids [4], and FDTD model refers to literature [5] for simulation. In the studied band, the real part of the complex refractive index of SiO_2_ is between 1.34 and 1.42, and the difference is only 0.07. On the other hand, the maximum value of imaginary part is less than 0.004 in Palik's handbook, so imaginary part of SiO_2_ complex refractive index for FDTD model is less than 10^-3^ and basically unchanged. Therefore, it is considered reasonable to take the refractive index of SiO_2_ as 1.39 for theoretical calculation.


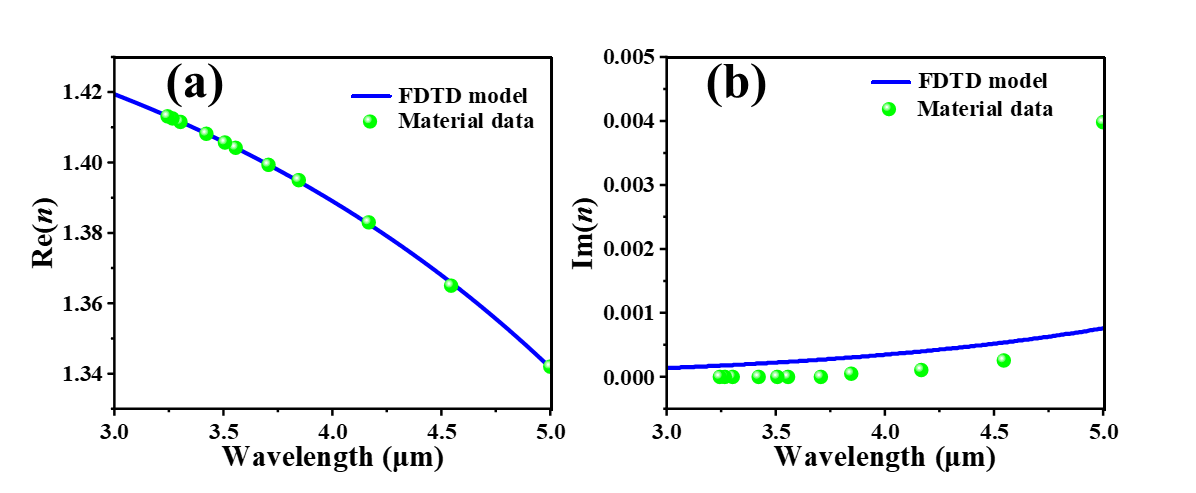


Fig. S2. The complex refractive index data of SiO_2_ for (a) real part and (b) imaginary part.

Supplementary Note 3

**The comprehensive fabrication process flow of MEMS-based meta-absorber.**

Fig. S3 shows two comprehensive fabrication process flows along AB line in Fig. S3(a). Figs. S3(b-j) show a surface micromachining process based on isotopically etching with the released etching holes. Figs. S3(k) and (l) show a bulk micromachining process based on anisotropically etching through DRIE process without the released etching holes.

For surface micromachining process, a SiO_2_ thin-film is first deposited on the surface of (100) n-type silicon (Si) substrate by using inductively coupled plasma chemical vapor deposition (ICP-CVD) process (Fig. S3(b)). Second, the lower layer of ETA and the location of infrared (IR) detector wires are patterned by using two photolithography and reactive ion etching (RIE) processes (Figs. S3(c) and (d)). Third, the upper layer of ETA and the bottom electrode of IR detector are defined by using electron-beam evaporation Al thin-film and lift-off processes (Fig. S3(e)). Fourth, a 300-nm-thick pyroelectric material (PM) layer, such as zinc oxide, is deposited and patterned by using sputtering and RIE processes (Fig. S3(f)), respectively. Fifth, the top electrode of IR detector and the reflective layer of meta-absorber are defined by using electron-beam evaporation 300-nm-thick Al thin-film and lift-off processes (Fig. S3(g)). Sixth, a SiO_2_ thin-film with the thickness of 1.60 μm is deposited and patterned by using ICP-CVD and RIE processes (Fig. S3(h)). Seventh, the 10 nm Au/5 nm Ti (adhesive layer) are deposited and patterned by using electron-beam evaporation and lift-off processes (Fig. S3(i)). Finally, the whole device is released by using ICP-RIE isotopically Si substrate process (Fig. S3(j)). As the mismatches of thermal expansion coefficients of Al and SiO_2_ layers, the bimaterial cantilevers and meta-absorber can be deflected upward to the out-of-plane direction.

For bulk micromachining process, when the device pattern is defined on the front side of Si substrate (Fig. S3(i)), the back side of Si substrate can be anisotropically etched through DRIE process to form the back side cavity (Fig. S3(k), the front side is protected by photoresist), and then etching Si substrate from front side to release the whole device (Fig. S3(l)).

The role of the released etching holes is to ensure that the large panel structure of meta-absorber can be completely released during the ICP-RIE isotopically process. By reasonably designing the number, size, and location of the released etching holes, the continuous surface and optical performances of the MIM cavity will not be affected, and the proposed meta-absorber can work normally. For example, as shown in Fig. S4(a), the defined releasing holes will not damage the optical performances of the device in literature [6]. On the other hand, as shown in Fig. S4(b), exploring a new etching process, such as dry isotropic etching of Si in etching gas of XeF_2_, that can also realize the releasing devices with large panel from the Si substrate without etching holes [7].


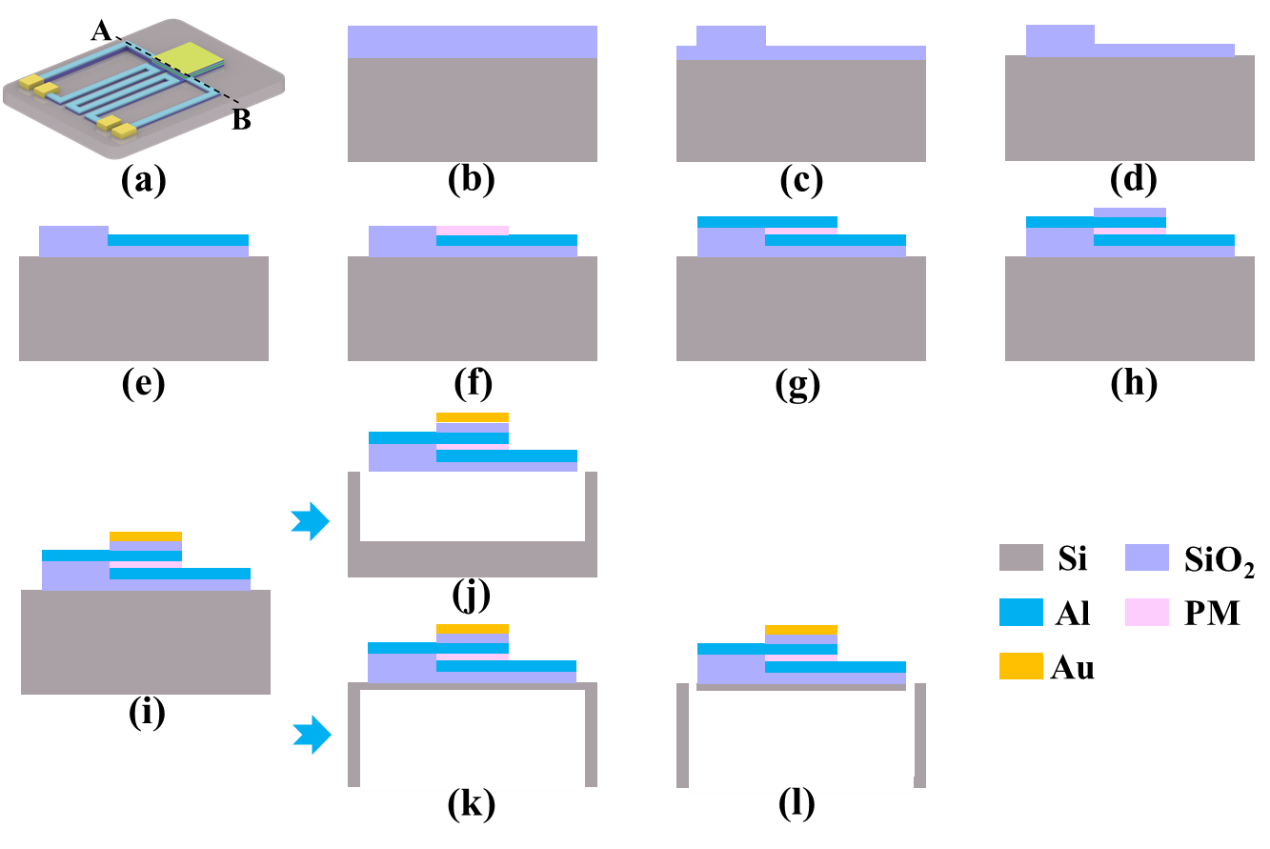


Fig. S3. The fabrication process flow of MEMS-based meta-absorber. (a) Schematic drawing of MEMS-based meta-absorber. (b) A SiO_2_ thin-film is deposited on the surface of (100) n-type silicon (Si) substrate by using ICP-CVD process. (c) and (d) SiO_2_ thin-film is patterned sequentially by using photolithography and RIE processes. (e) The bottom Al thin-film is deposited and patterned by using electron-beam evaporation and lift-off processes. (f) The 300-nm-thick PM thin-film is deposited and patterned by using sputtering and RIE processes. (g) The top 300-nm-thick Al thin-film is deposited and patterned by using electron-beam evaporation and lift-off processes. (h) The SiO_2_ thin-film with the thickness of 1.60 μm is deposited and patterned by using ICP-CVD and RIE processes. (i) The 10 nm Au/5 nm Ti (adhesive layer) are deposited and patterned by using electron-beam evaporation and lift-off processes. (j) The whole device is released by using ICP-RIE isotopically Si substrate process. (k) The back side of sample is anisotropically etched through DRIE process. (l) The font side of sample is anisotropically etched through DRIE process to release the whole device.


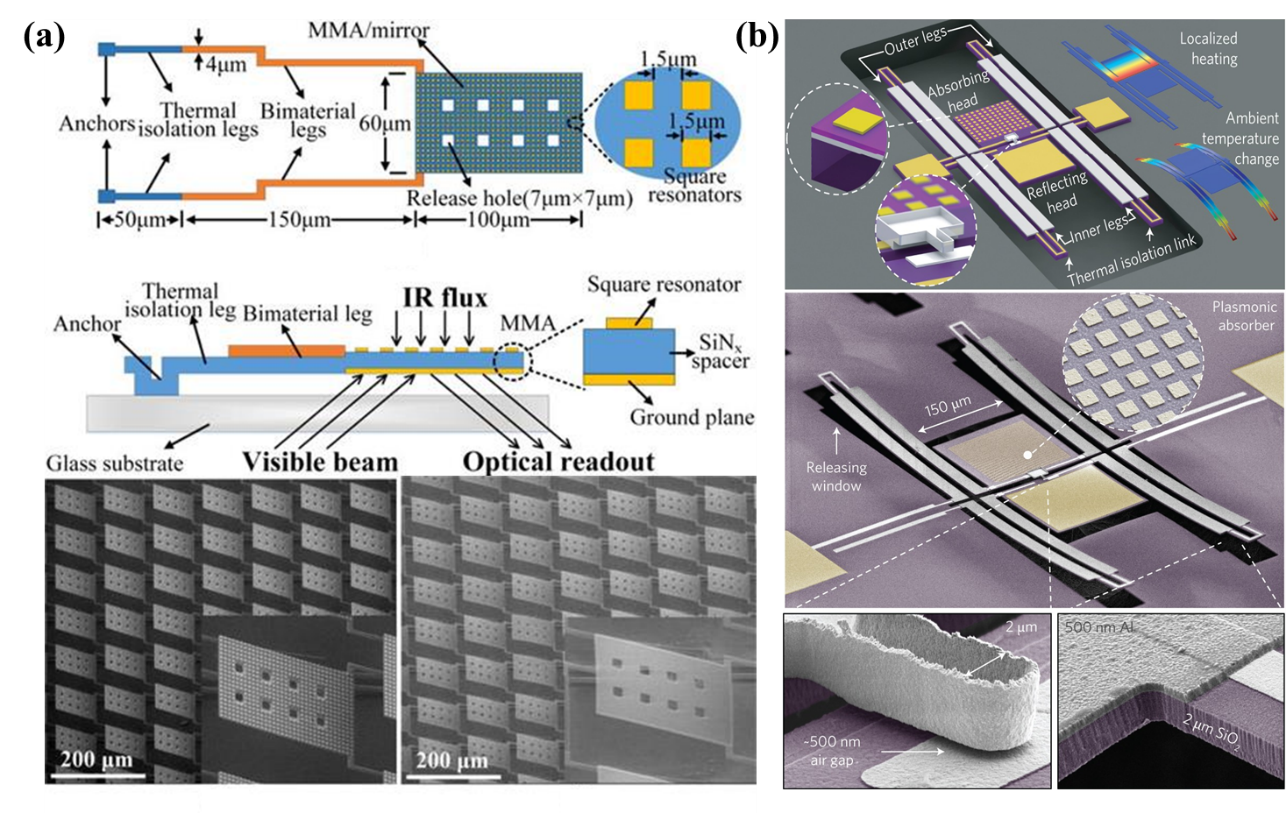


Fig. S4. (a) Metamaterial enhanced MEMS thermal bimaterial cantilever infrared focal plane array [6]. (b) Zero-power digitized infrared detector based on plasmonically enhanced MEMS thermal photoswitch [7].

Supplementary Note 4

**The ultra-low power consumption configuration of MEMS-based meta-absorber.**

The required maximum applied voltage (*U_m_*) for the proposed MEMS-based meta-absorber is 0.345 volts, which is a fairly low driving voltage. We simulated the current flow (*I*) and calculated the consumed power (*P*) as shown in Fig. S5. The current flow increases from 0 mA to 518 mA when the applied voltage increases from 0 volt to 0.34 volts, which means that the maximum current flow does not exceed the order of 1 A. The relationship between current flow and voltage is rather linear (the linear correction coefficient *R*^2^ = 0.9999), indicating that it has linear resistance characteristic. Moreover, the consumed power is calculated according to the physical relation *P* = *UI*. The maximum power consumption exhibited by the MEMS-based meta-absorber is less than 180 mW. The deformation power consumption of the proposed MEMS-based meta-absorber is calculated to be about 0.5°/mW according to Eq. (10). These values are enough to prove the low power consumption configuration of the MEMS-based meta-absorber under the condition of meeting the large deflection angle.

Fig. S5. The corresponding relationship of current flow (*I*) and power along with the applied DC bias voltage.

Supplementary Note 5

**The necessary collimation accuracy for NDIR gas sensing in Fig. 7.**

The necessary accuracy of the collimation angle is explained that the allowable collimation error angle under the condition of limit resolution, that is, the allowable collimation angle deviation when deviating from the optimal incident angle but still detecting the target gas. It is related to the angular resolution, which is defined as the variation of resonant wavelength at unit angle, which can be obtained by differentiating Eq. (4). For more specific explanation, we take *P*-polarized light as an example to deduce. According to the Eq. (4) and linear relation of Fig. 3(c), we can obtain the relationship between the resonance of *P*-polarized light and incident angle as follow.

$$\begin{aligned} \text{λ}_{\text{rP}}\text{ = 3.7438}\sqrt{\text{1–}\left( \frac{\text{sin(}\text{θ}_{\text{i}}\text{)}}{\text{n}_{\text{d}}} \right)^{\text{2}}}+\text{0.8571}\#\left( \text{S8} \right) \end{aligned}$$

By taking the derivative of Eq. (S8), we can obtain the angular resolution for *P*-polarized light as follow.

$$\begin{aligned} \left| \frac{\text{d}\text{λ}_{\text{rP}}}{\text{d}\text{θ}_{\text{i}}} \right|\text{=}\frac{\text{3.7438}}{\text{2}\text{n}_{\text{d}}^{\text{2}}}\frac{\text{sin(}\text{2}\text{θ}_{\text{i}}\text{)}}{\sqrt{\text{1}\text{–}\left( \frac{\text{sin(}\text{θ}_{\text{i}}\text{)}}{\text{n}_{\text{d}}} \right)^{\text{2}}\text{.}}}.\#\text{(S9)} \end{aligned}$$

where *λ_rP_* is resonant wavelength for *P*-polarized light, *n_d_* is refractive index of dielectric layer, and *θ_i_* is the incident angle. To simplify the calculation, we approximate the refractive index *n_d_* of SiO_2_ as 1.39 according to Supplementary Note 2. The distribution of angular resolution for *P*-polarized light is shown in Fig. S6(a). The angular resolution for *P*-polarized light is not a constant, and it increases first and then decreases. The maximum angular resolution is 20 nm/° when the incident angle is 50°. It means that when the incident angle is around 50°, the resonant wavelength shifts by about 20 nm for every 1° change of it. On the other hand, the high selectivity is approximately related to the FWHM values of the absorption spectrum according to Rayleigh Criterion. For *P*-polarized light, and the maximum FWHM value is 112 nm except for the incident angle of 80° with a large divergence according to Fig. 3(c). Therefore, to the wavelength spreads of 112 nm with *P*-polarized light for mixed gas identification, the maximum allowable angular collimation error is 112 nm/20 nm/° = 5.6°. This value will increase at other incident angles due to the decrease of angular resolution. For *S*-polarized light, the distribution of angular resolution can be obtained through a similar process according to the Eq. (4) and linear relation of Fig. 3(d), as shown in Fig. S6(b). The maximum angular resolution is 22 nm/° when the incident angle is 50°. It means that when the incident angle is around 50°, the resonant wavelength shifts by about 22 nm for every 1° change of it. For *S*-polarized, and the maximum FWHM value is 37 nm according to Fig. 3(d). Therefore, to the wavelength spreads of 37 nm with *S*-polarized light for mixed gas identification, the maximum allowable angular collimation error is 37 nm/22 nm/° ≈ 1.68°.

Although we calculate the theoretical angular resolution and the theoretical maximum allowable angular collimation error, it may not be the case in the actual gas sensing application. The reason is that the limit resolution only occurs when the incident angle is around 50° (corresponding resonances are *λ_rP_* = 3.99 μm and *λ_rS_* = 3.93 μm, respectively) and the differences between the characteristic absorption peaks of the two gases are only 112 nm for *P*-polarized light and 37 nm for *S*-polarized light. The difference of absorption peaks of common gases is greater than these values (112 nm and 37 nm) and the angular resolution decreases at other incident angles, which will increase the maximum allowable angular collimation error. In addition, as shown in Fig. 4, the absorption of gases does not exist only in one wavelength, but has a spectral width, which will also increase the maximum allowable angular collimation error. For example, the absorption of CO_2_ gas is strong in the wavelength range of 4.20 μm to 4.33 μm, so the estimated maximum allowable collimation error can be about 9° at the incident angle of 36° for *P*-polarized light and 7° at the incident angle of 34° for *S*-polarized light, respectively.


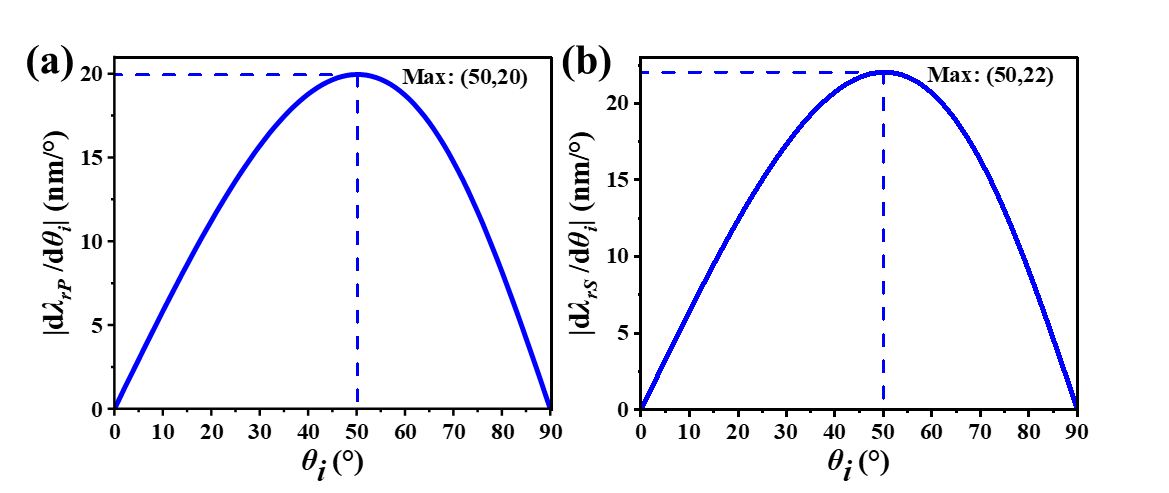


Fig. S6. The distribution of angular resolution for (a) *P*-polarized light and (b) *S*-polarized light, respectively.

Supplementary Note 6

**The brief explanation to the influence of polarization of light on absorption spectra.**

Fig. S7 shows the simulated and measured normalized absorption spectra of meta-absorber. The results of measurement are consistent with Fig. 8(c), which is obtained by the measured of unpolarized light. The simulated results of Fig. S7(a) are the intermediate values of *P*-polarized and *S*-polarized light in Figs. 8(a) and (b), which is calculated by formula 0.5*(*A_P_*+*A_S_*) in the same angle and the normalized. Here, *A_P_* and *A_S_* represent the absorptance of *P*-polarized and *S*-polarized light, respectively. This is used to approximate completely unpolarized light, that is, half of the power is delivered as *P*-polarized light and the other half is delivered as *S*-polarized light. As shown in Fig. S7(a), the results of simulation and measurement have similar intensity trend and angle dispersion characteristic. Due to the different resonance characteristics of *P*-polarized and *S*-polarized light, the FWHM values of absorption peak are increased by the influence of mixed polarization. This is conducive to increase the allowable collimation error angle, but at the expense of gas sensing selectivity. It may be necessary to make trade-offs in practical application. A light source with a certain polarization state (*P*- or *S*-) is tended to be used in practical application, because this will greatly improve the sensitivity and accuracy of gas sensing. The measured resonant wavelengths are deviated from the simulation due to the artificial error of the actual incident angle and the manufacturing process deviation of the thickness and quality of SiO_2_ and Au thin-film. To illustrate this point, we simulated the absorptance of the meta-absorber with dielectric layer thickness of 1.51 μm according to Eqs. (1) and (2) and the measured absorption peak at incident angle 30°, the results are shown in Fig. S7(b). Similarly, the simulated results are the intermediate values of *P*-polarized and *S*-polarized light. The measured and simulated resonances and intensities have a better correspondence when the thickness error decreases. This confirms our point of view.


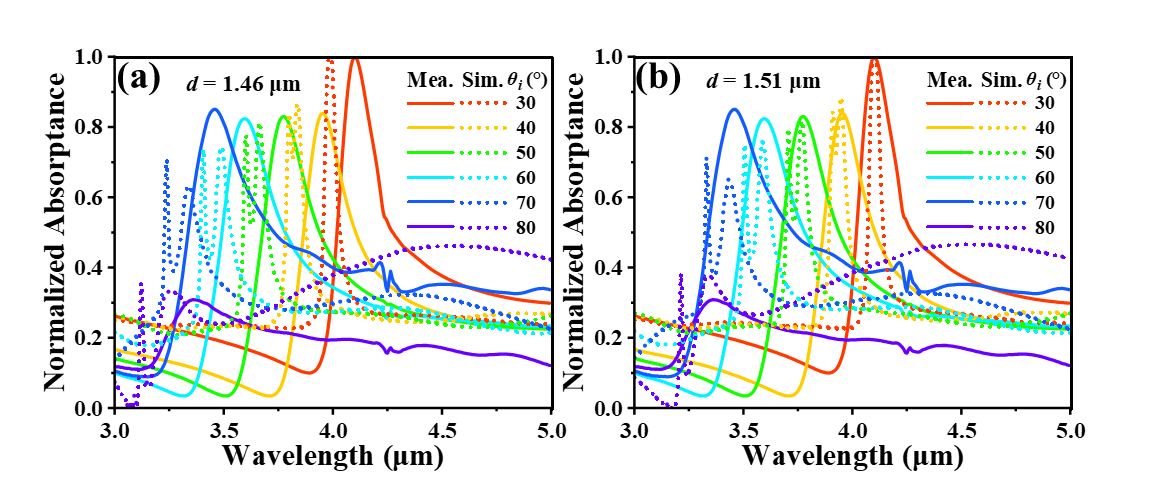


Fig. S7. The simulated and measured normalized absorption spectra of meta-absorber with the dielectric layer thickness of (a) 1.46 μm and (b) 1.51 μm.

Supplementary Note 7

**The influence of the thickness of Au thin-film atop on absorption spectra of meta-absorber.**

Figs. S8(a) and (b) show the influence of the thickness of Au thin-film on the absorption spectra of meta-absorber when the illumination source is normal incidence onto the planar surface and the thickness of SiO_2_ layer is 1.46 μm. When the thickness of Au thin-film is changed from 5 nm to 15 nm, the absorption peak is blue-shifted from the wavelength of 4.31 μm to 4.19 μm while the FWHM values decrease from 88 nm to 20 nm. The absorptance begins to decrease when the thickness of Au is more than 7 nm. Therefore, the irreversible red-shift of the resonance peak occurs when the evaporated Au layer is too thin.


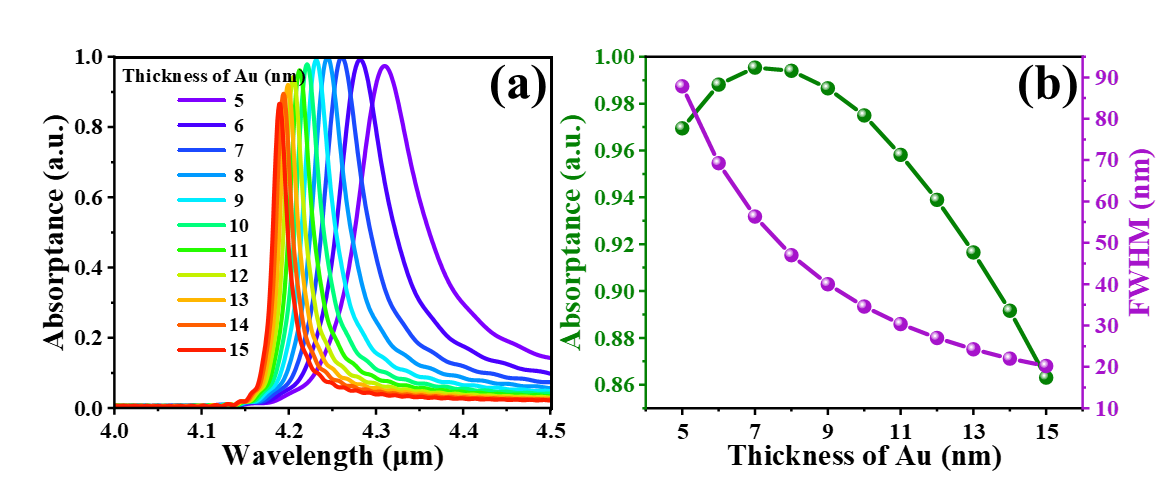


Fig. S8. (a) Absorption spectra of the meta-absorber with different thicknesses of Au thin-film when the illumination source is normal incidence onto the planar surface. (b) The corresponding relationship of absorptance and FWHM values with the thicknesses of Au thin-film.

Supplementary Note 8

**The CO_2_ testing process of meta-absorber.**

The testing set-up is shown in Fig. 9(a). The FTIR spectrometer-IR microscopic imaging combined instrument (model: Vertex70-Hyperion3000) is used for testing, and all measurements are launched under the condition of normal incidence of light source. To simplify the test, the air background is used as a reference since the vacuum condition cannot be set simply. And only the absorption of CO_2_ in the air is considered in the studied band. The natural logarithm is used to analyze and discuss the results.

1. The measurement of reference spectrum of air background.

As shown in Fig. S9(a), the reflection spectrum of air background is measured by aluminum reflective mirror (to facilitate the explanation, the light source is drawn as inclined in the figure), and the result is shown in Fig. S9(b). The reflectance of the Al reflective mirror is assumed to be 1. The decrease of reflectance at 4.25 μm is caused by the inherent CO_2_ absorption in the air. The reflectance *R*_1_ satisfies

$$\begin{aligned} \text{R}_{\text{1}}\text{ = }\frac{\text{I}_{\text{1}}}{\text{I}_{\text{0}}}\text{ =}\exp\left( -\varepsilon\text{c}_{\text{1}}\text{∙2}\text{L} \right)\#\left( \text{S10} \right) \end{aligned}.$$

where $\text{ε}$ is the molar absorption coefficient of CO_2_ gas, *c*_1_ is the concentration of CO_2_ gas in the air, and *L* is the path length. Since the reflectance of the Al reflective mirror is 1, the absorptance of the air background can be calculated as *A*_1_ = 1 - *R*_1_ (Fig. S9(c)), which corresponds to the red curve in Fig. 9(b).


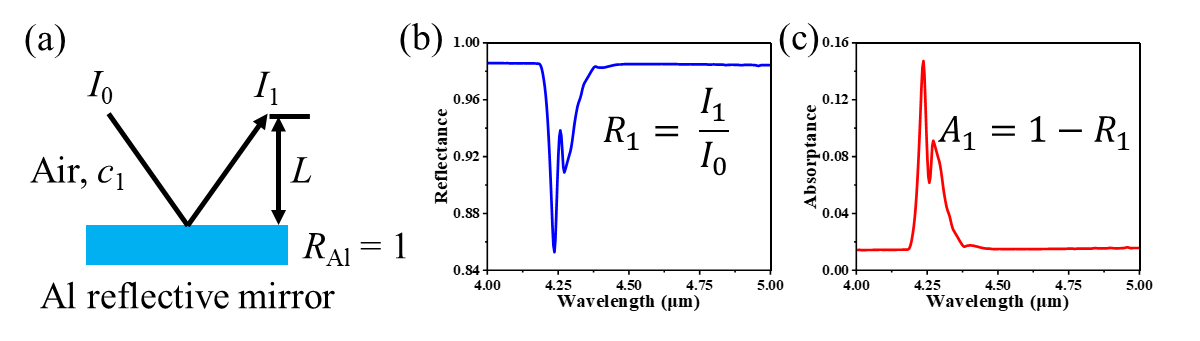


Fig. S9. The measurement of reference spectrum of air background. (a) The schematic diagram of measurement. The measured reflection (b) and absorption (c) spectra of air background.

2. The measurement of absorption spectrum of meta-absorber.

As shown in Fig, S10(a), the aluminum mirror is replaced by meta-absorber, and the reflection spectrum is measured again, and the result is shown in Fig. S10(b). The reflectance *R*_2_ satisfies

$$\begin{aligned} \text{R}_{\text{2}}\text{ = }\frac{\text{I}_{\text{2}}}{\text{I}_{\text{0}}}\text{ =}\exp\left( -\text{ε}\text{c}_{\text{1}}\text{L} \right)\text{∙}\text{R}_{\text{m}}\text{∙}\exp\left( -\text{ε}\text{c}_{\text{1}}\text{L} \right)\text{ =}\exp\left( -\text{ε}\text{c}_{\text{1}}\text{∙2}\text{L} \right)\text{∙}\text{R}_{\text{m}}\text{ = }\text{R}_{\text{1}}\text{R}_{\text{m}}\#\left( \text{S11} \right) \end{aligned}.$$

Here, *R*_m_ is the reflectance of the meta-absorber. Since the transmittance of the meta-absorber is 0, as shown in Fig. S10(c), the absorptance of the meta-absorber can be calculated as

$$\begin{aligned} \text{A}_{\text{m}} = \text{1}-\text{R}_{\text{m}} = \text{1}-\frac{\text{R}_{\text{2}}}{\text{R}_{\text{1}}}\#\left( \text{S12} \right). \end{aligned}$$

It corresponds to the green curve in Fig. 9(b).


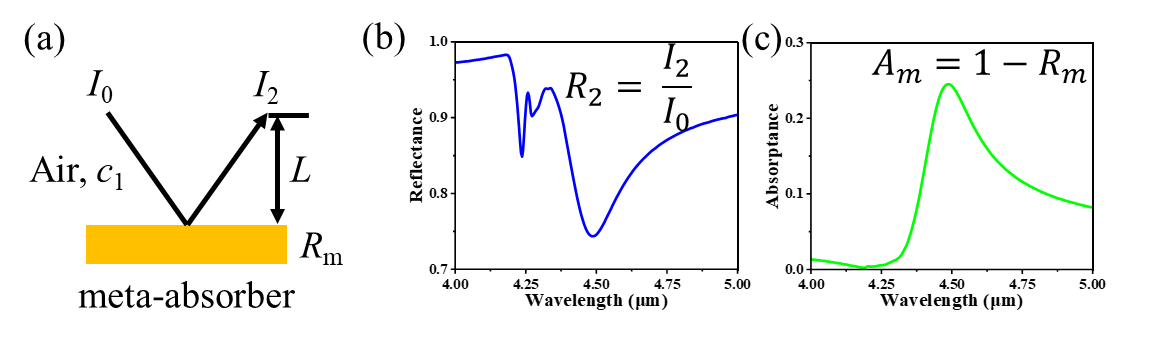


Fig. S10. The measurement of absorption spectrum of meta-absorber. (a) The schematic diagram of measurement. (b) The measured reflection spectrum of meta-absorber in the air. (c) The measured absorption spectrum of meta-absorber.

3. The measurement of absorption spectrum of introduced high concentration CO_2_ gas.

The Al reflective mirror is placed in the sample cavity of gas cell, and high concentration CO_2_ is introduced, as shown in Fig. S11(a). We neglect the reflection of the window surface of the gas cell. The reflection spectrum is measured again, and the result is shown in Fig. S11(b). The reflectance *R*_3_ satisfies

$$\begin{aligned} \text{R}_{\text{3}}\text{ = }\frac{\text{I}_{\text{3}}}{\text{I}_{\text{0}}}\text{ = }\exp\left( -\text{ε}\text{c}_{\text{1}}\text{∙2}\text{L}_{\text{1}} \right)\text{∙}\exp\left( -\text{ε}\text{c}_{\text{2}}\text{∙2}\text{L}_{\text{2}} \right)\#\left( \text{S13} \right) \end{aligned}.$$

Here, *c*_2_ and *L*_2_ are the concentration of CO_2_ gas in the cavity and the length of cavity, respectively. In our testing set-up, $\text{L}_{\text{1}}\text{ ≈ }\text{L}\text{ }\text{≫}\text{ }\text{L}_{\text{2}}$, so the following relation can be obtained approximately,

$$\begin{aligned} \text{R}_{\text{3}}\text{ ≈}\exp\left( -\text{ε}\text{c}_{\text{1}}\text{∙2}\text{L} \right)\text{∙}\exp\left( -\text{ε}\text{c}_{\text{2}}\text{∙2}\text{L}_{\text{2}} \right)\text{ =}{\text{ }\text{R}}_{\text{1}}\text{R}_{\text{CO}_{\text{2}}}\#\left( \text{S14} \right) \end{aligned}.$$

Here, $\text{R}_{\text{CO}_{\text{2}}}$ is the reflection spectrum caused by CO_2_ gas in the cavity. Therefore, as shown in Fig. S11(c), the absorptance of the high concentration CO_2_ can be calculated as

$$\begin{aligned} \text{A}_{\text{CO}_{\text{2}}}=\text{1}-\text{R}_{\text{CO}_{\text{2}}}=\text{1}-\frac{\text{R}_{\text{3}}}{\text{R}_{\text{1}}}\#\left( \text{S15} \right). \end{aligned}$$

It corresponds to the cyan curve in Fig. 9(b).


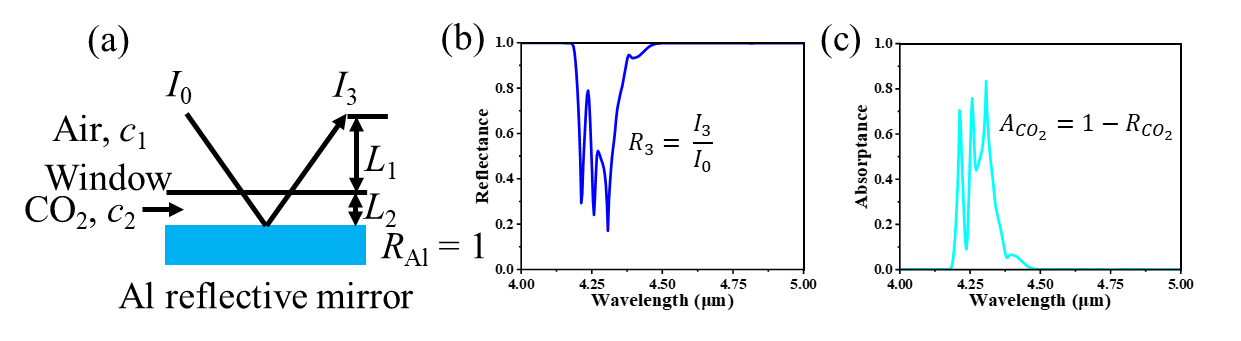


Fig. S11. The measurement of absorption spectrum of introduced high concentration CO_2_ gas. (a) The schematic diagram of measurement. (b) The measured reflection spectrum as the high concentration CO_2_ gas is introduced. (c) The measured absorption spectrum of high concentration CO_2_.

4. The measurement of absorption spectrum of the meta-absorber under high concentration CO_2_ gas.

As shown in Fig. S12(a), the Al reflective mirror in the gas cell is replaced by the meta-absorber, and high concentration CO_2_ gas is introduced and the reflection spectrum is measured again. The result is shown in Fig. S12(b). The reflectance *R*_4_ satisfies

$$\begin{aligned} \text{R}_{\text{4}}\text{ = }\frac{\text{I}_{\text{4}}}{\text{I}_{\text{0}}}\text{ =}\exp\left( -\text{ε}\text{c}_{\text{1}}\text{∙2}\text{L}_{\text{1}} \right)\text{∙}\text{R}_{\text{m}}\text{∙}\exp\left( -\text{ε}\text{c}_{\text{3}}\text{∙2}\text{L}_{\text{2}} \right)\#\left( \text{S16} \right) \end{aligned}.$$

Here, *c*_3_ is the concentration of CO_2_ introduced for the second time. Similarly, the following relation can be obtained approximately,

$$\begin{aligned} \text{R}_{\text{4}}\text{ ≈ }\exp\left( -\text{ε}\text{c}_{\text{1}}\text{∙2}\text{L} \right)\text{∙}\text{R}_{\text{m}}\text{∙}\exp\left( -\text{ε}\text{c}_{\text{3}}\text{∙2}\text{L}_{\text{2}} \right)\text{= }\text{R}_{\text{1}}\text{R}_{\text{m}\text{, }\text{CO}_{\text{2}}}\#\left( \text{S17} \right) \end{aligned}.$$

Here, $\text{R}_{\text{m}\text{, }\text{CO}_{\text{2}}}$ is the reflection spectrum caused by the meta-absorber under high concentration CO_2_ gas in the cavity. Therefore, as shown in Fig. S12(c), the absorptance of the meta-absorber under high concentration CO_2_ gas can be calculated as

$$\begin{aligned} \text{A}_{{\text{m}\text{,CO}}_{\text{2}}}=\text{1}-\text{R}_{\text{m}\text{,}\text{CO}_{\text{2}}}=\text{1}-\frac{\text{R}_{\text{4}}}{\text{R}_{\text{1}}}\#\left( \text{S18} \right). \end{aligned}$$

It corresponds to the purple curve in Fig. 9(b).


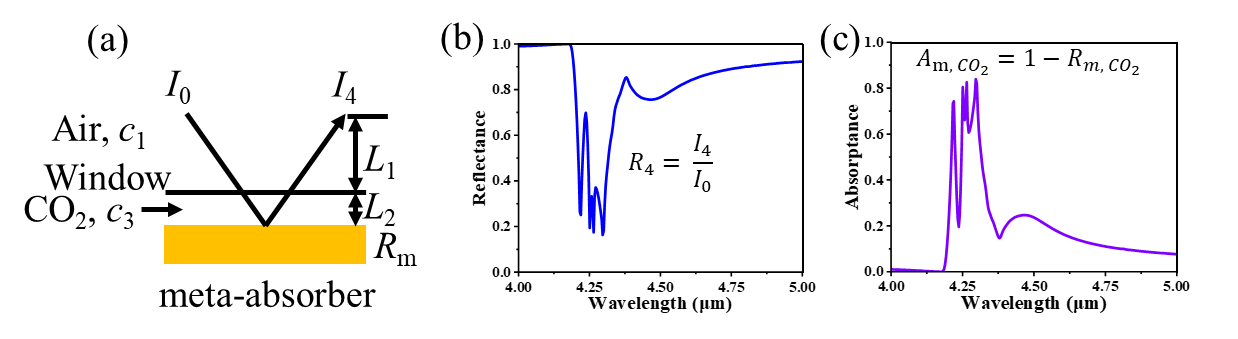


Fig. S12. The measurement of absorption spectrum of the meta-absorber under high concentration CO_2_ gas. (a) The schematic diagram of measurement. (b) The measured reflection spectrum of meta-absorber as the high concentration CO_2_ gas is introduced. (c) The measured absorption spectrum of meta-absorber under high concentration CO_2_ gas.

To sum up, Fig. 9(b) is obtained by combining Fig. S9(c), Fig. S10(c), Fig. S11(c) and Fig. S12(c). It is worth noting that it is difficult to ensure that the concentrations (*c*_2_ and *c*_3_) of CO_2_ gas injected twice are completely consistent due to the lack of more advanced equipment the influence of spectrometer state. Therefore, the cyan and purple curves in Fig. 9(b) are slightly different around the wavelength of 4.25 μm. Before the integrated an IR detector, it is difficult for us to judge its specific concentration since the absorption of CO_2_ gas with different concentrations fluctuates greatly at the wavelength of 4.25μm. However, our results are sufficient to illustrate the influence of CO_2_ gas on the absorption characteristic of the meta-absorber. It is reasonable to believe that the proposed meta-absorber is effective in NDIR gas sensing application after integrating the IR detector.

Supplementary Note 9

**The absorption peaks** **analysis of the spectra in Fig. 9(b).**

From the absorption spectrum of Fig. S9(c), two obvious absorption peaks are observed at the wavelengths of 4.24 μm and 4.27 μm, and a valley is formed at the wavelength of 4.26 μm. The result comes from the conventional absorption of low concentration CO_2_ gas in the air. When high concentration CO_2_ gas is introduced, three absorption peaks at the wavelengths of 4.21 μm, 4.26 μm and 4.29 μm are observed in the absorption spectrum of Fig. S11(c), and four absorption peaks at the wavelengths of 4.21 μm, 4.25 μm, 4.26 μm, and 4.29 μm are observed in the absorption spectrum of Fig. S12(c). The reason for the formation of multiple peaks is that the infrared absorption spectrum is supersaturated when the CO_2_ gas with high concentration is introduced, which leads to the splitting of absorption peaks. To illustrate this phenomenon, Table S1 shows the distribution of absorption peaks and valleys of *A*_1_, $\text{A}_{\text{CO}_{\text{2}}}$ and $\text{A}_{{\text{m}\text{,CO}}_{\text{2}}}$. The absorption peaks of high concentration CO_2_ gas are just distributed on both sides of the absorption peaks of low concentration CO_2_ gas in the air, and the absorption valleys are just located at the absorption peaks of low concentration CO_2_ gas in the air. These fully show that the multiple peaks formed in Figs. S11(c) and S12(c) are the result of the splitting of absorption peaks in Fig. S9(c) due to the supersaturation of absorption spectrum under the condition of high concentration CO_2_ gas. Meanwhile, only three peaks were observed in Fig. S11(c), which may be because the split absorption peaks merged or were too close to distinguish.

Table S1. The distribution of absorption peaks and valleys of *A*_1_, $\text{A}_{\text{CO}_{\text{2}}}$ and $\text{A}_{{\text{m}\text{,CO}}_{\text{2}}}$.

| Wavelength (μm) | 4.21 | 4.24 | 4.25 | 4.26 | 4.27 | 4.29 |
| --- | --- | --- | --- | --- | --- | --- |
| *A*_1_ (*c*_1_) | - | Peak | - | Valley | Peak | - |
| $\text{A}_{\text{CO}_{\text{2}}}$ (*c*_2_) | Peak | Valley | - | Peak | Valley | Peak |
| $\text{A}_{\text{m}\text{, }\text{CO}_{\text{2}}}$ (*c*_3_) | Peak | Valley | Peak | Peak | Valley | Peak |

Supplementary Note 10

**The simple theoretical analysis of meta-absorber applying in NDIR gas sensing.**

In Supplementary Note 8, we use absorption spectra to show the influence of CO_2_ gas on the absorption spectrum of the meta-absorber. However, the light energy absorbed by the meta-absorber is the standard to evaluate the sensing for NDIR gas sensing. To simplify the derivation process, we simply use light intensity (*I*) as light energy here, which will not affect the rationality of the results. According to Eq. (S10), the light energy absorbed by CO_2_ gas (*I*_air_) in the air is as follows,

$$\begin{aligned} \text{I}_{\text{air}}\text{ = }\text{I}_{\text{0}}-\text{I}_{\text{1}}\#\left( \text{S19} \right) \end{aligned}.$$

Then, according to Eq. (S11), the total light energy absorbed by CO_2_ gas in the air and the meta-absorber can be expressed by

$$\begin{aligned} \text{I}_{\text{air and meta}}\text{ = }\text{I}_{\text{0}}-\text{I}_{\text{2}}=\text{I}_{\text{0}}-\text{I}_{\text{0}}\text{R}_{\text{1}}\text{R}_{\text{m}}=\text{I}_{\text{0}}-\text{I}_{\text{1}}\text{R}_{\text{m}}\#\left( \text{S20} \right) \end{aligned}.$$

Therefore, the light energy absorbed by the meta-absorber (*I*_meta, 1_) can be expressed as

$$\begin{aligned} \text{I}_{\text{meta}\text{, }\text{1}}\text{ = }\text{I}_{\text{air and meta}}-\text{I}_{\text{air}}=\text{I}_{\text{1}}\left( \text{1}-\text{R}_{\text{m}} \right)=\text{ }\text{I}_{\text{0}}\text{A}_{\text{m}}\text{∙ }\text{exp} \left( -\varepsilon\text{c}_{\text{1}}\text{∙2}\text{L} \right)\#\left( \text{S21} \right) \end{aligned}.$$

It can be seen from Eq. (S21) that the light energy absorbed by the meta-absorber has a form like Beer-Lambert law, and it is not only related to its own absorptance, but also related to the concentration of CO_2_ gas in the air (the path length *L* is constant). The higher the concentration of CO_2_ gas in the air, the less light energy the meta-absorber obtains, which results in the weakening of the output voltage signal after integrating the IR pyroelectric detector.

Similarly, according to Eq. (S13), the light energy absorbed by the introduced high concentration CO_2_ gas ($\text{I}_{\text{CO}_{\text{2}}}$) can be expressed as

$$\begin{aligned} \text{I}_{\text{CO}_{\text{2}}}\text{ = }\text{I}_{\text{0}}-\text{I}_{\text{3}}\#\left( \text{S22} \right) \end{aligned}.$$

Assuming that the concentration of CO_2_ gas introduced twice is basically the same ($c_{3}\approx c_{2}\text{)}$, according to Eq. (S16), the total light intensity absorbed by the introduced high concentration CO_2_ gas and the meta-absorber can be expressed by

$$\begin{aligned} \text{I}_{\text{CO}_{\text{2}}\text{ and meta}}\text{ = }\text{I}_{\text{0}}-\text{I}_{\text{4}}=\text{I}_{\text{0}}-\text{I}_{\text{0}}\exp\left( -\text{ε}\text{c}_{\text{1}}\text{∙2}\text{L}_{\text{1}} \right)\text{∙}\text{R}_{\text{m}}\text{∙}\exp\left( -\text{ε}\text{c}_{\text{3}}\text{∙2}\text{L}_{\text{2}} \right)\approx\text{I}_{\text{0}}-\text{I}_{\text{3}}\text{R}_{\text{m}}\#\left( \text{S23} \right). \end{aligned}$$

In this case, the light energy absorbed by the meta-absorber (*I*_meta, 2_) can be expressed as

$$\begin{aligned} \text{I}_{\text{meta, 2}}\text{ = }\text{I}_{\text{CO}_{\text{2}}\text{ and meta}}-\text{I}_{\text{CO}_{\text{2}}}=\text{I}_{\text{3}}\left( \text{1}-\text{R}_{\text{m}} \right)=\text{ }\text{I}_{\text{0}}\text{A}_{\text{m}} \exp\left( -\text{ε}\text{c}_{\text{1}}\text{∙2}\text{L}_{\text{1}} \right)\exp\left( -\text{ε}\text{c}_{\text{2}}\text{∙2}\text{L}_{\text{2}} \right) \\ \approx\text{I}_{\text{meta, 1}}\cdot\exp\left( -\text{ε}\text{c}_{\text{2}}\text{∙2}\text{L}_{\text{2}} \right)\#(\text{S24}). \end{aligned}$$

The light energy absorbed by the meta-absorber for the second time is $\exp\left( -\text{ε}\text{c}_{\text{2}}\text{∙2}\text{L}_{\text{2}} \right)$ times that of the first time, which indicates that the light energy absorbed by the meta-absorber decreases due to the introduction of CO_2_ gas. If $\text{I}_{\text{meta, 1}}$ is taken as the reference (as there is no vacuum condition), the responsivity of CO_2_ gas or the degree of signal change can be approximated as

$$\begin{aligned} \frac{\text{I}_{\text{meta, 1}}-\text{I}_{\text{meta, 2}}}{\text{I}_{\text{meta, 1}}}\boldsymbol{=}\text{1}-\exp\left( -\text{ε}\text{c}_{\text{2}}\text{∙2}\text{L}_{\text{2}} \right)\boldsymbol{=}\text{A}_{\text{CO}_{\text{2}}}\boldsymbol{\#}\left( \text{S25} \right) \end{aligned}\mathbf{.}$$

Eq. (S25) is basically consistent with that in literature [8,9]. According to Eq. (S25), the responsivity of CO_2_ gas or the degree of signal change is equal to the absorptance of the introduced CO_2_ gas ($\text{A}_{\text{CO}_{\text{2}}}$), which is quite reasonable. When CO_2_ gas is not introduced, *c*_2_ = 0 and $\text{A}_{\text{CO}_{\text{2}}}$= 0, so the responsivity of CO_2_ gas is equal to 0 and $\text{I}_{\text{meta, 2}}=\text{I}_{\text{meta, 1}}$. This means that the light energy absorbed by the meta-absorber at this time is the same as that when it is placed in the air (that is, no additional CO_2_ gas is introduced). On the other hand, when CO_2_ gas with extremely high concentration is introduced, *c*_2_ = ∞ and $\text{A}_{\text{CO}_{\text{2}}}$= 1 (at the absorption wavelength), so the responsivity of CO_2_ gas reaches 1 and $\text{I}_{\text{meta, 2}}=0$. This means that no energy is absorbed by the meta-absorber, because all energy is dissipated by the high concentration CO_2_ gas.

To show the quantitative relationship more clearly, based on the absorptance of CO_2_ at the wavelength of 4.26 μm in Fig. S11(c) (the splitting of the peak is ignored because we only use it as a benchmark for verification), we draw the relationship between CO_2_ responsivity and CO_2_ concentration as shown in Fig. S13 below according to Eq. (S25). The higher the concentration of the CO_2_ gas, the greater the responsivity. Therefore, the feasibility of sensing has preliminarily confirmed in theory.

Fig. S13. The relationship between CO_2_ responsivity and CO_2_ concentration.

**Supplementary References:**

1. B. J. Lee, Z. M. Zhang, Design and fabrication of planar multilayer structures with coherent thermal emission characteristics, J. Appl. Phys. **100**, 063529 (2006).
2. R. G. Greenler, Infrared Study of Adsorbed Molecules on Metal Surfaces by Reflection Techniques. J. Chem. Phys. **44**, 310-315 (1966).
3. A. D, Rakić. Algorithm for the determination of intrinsic optical constants of metal films: application to aluminum, Appl. Opt. **34**, 4755-4767 (1995).
4. E. Palik and G. Ghosh, Handbook of Optical Constants of Solids, Academic, (1998).
5. J. Kischkat, S. Peters, B. Gruska, M. Semtsiv, M. Chashnikova, M. Klinkmüller, O. Fedosenko, S. Machulik, A. Aleksandrova, G. Monastyrskyi, Y. Flores, W. T. Masselink, Mid-infrared optical properties of thin films of aluminum oxide, titanium dioxide, silicon dioxide, aluminum nitride, and silicon nitride, Appl. Opt. **51**, 6789-6798 (2012).
6. W. Ma, Y. Wen, X. Yu, Y. Feng, Y. Zhao, Performance enhancement of uncooled infrared focal plane array by integrating metamaterial absorber, Appl. Phys. Lett. **106**, 111108 (2015).
7. Z. Qian, S. Kang, V. Rajaram, C. Cassella, N. E. McGruer, M. Rinaldi, Zero-power infrared digitizers based on plasmonically enhanced micromechanical photoswitches, Nature Nanotech. **12**, 969-973 (2017).
8. D. K. T. Ng, C. P. Ho, L. Xu, W. Chen, Y. H. Fu, T. Zhang, L. Y. Siow, N. Jaafar, E. J. Ng, Y. Gao, H. Cai, Q. Zhang, L. Y. T. Lee, NDIR CO_2_ gas sensing using CMOS compatible MEMS ScAlN-based pyroelectric detector, Sens. Actuators B Chem. **346**, 130437 (2021).
9. X. Tan, H. Zhang, J. Li, H. Wan, Q. Guo, H. Zhu, H. Liu, F. Yi, Non-dispersive infrared multi-gas sensing via nanoantenna integrated narrowband detectors, Nat. Commun. **11**, 5245 (2020).
